# An acid-based DES as a novel catalyst for the synthesis of pyranopyrimidines

**DOI:** 10.1038/s41598-023-45352-4

**Published:** 2023-10-21

**Authors:** Arezo Monem, Davood Habibi, Hadis Goudarzi

**Affiliations:** Department of Organic Chemistry, Faculty of Chemistry, Bu-Ali Sina University, Hamedan, 6517838683 Iran

**Keywords:** Chemistry, Materials science

## Abstract

Deep eutectic solvents have countless advantages over normal solvents, and in addition to complying with the principles of green chemistry, depending on their nature, they can also act as catalysts. The use of deep eutectic solvents as acid catalysts has several advantages such as non-toxicity, a catalytic effect similar to or higher than the acid itself, and the possibility of recovery and reuse without significant loss of activity. In this project, A novel deep eutectic solvent (MTPPBr–PCAT–DES) was prepared from a one-to-one mole ratio of methyltriphenyl-phosphonium bromide (MTPPBr) and 3,4-dihydroxybenzoic acid (PCAT = protocatechuic acid) and characterized by various techniques such as FT-IR, TGA/DTA, densitometer, eutectic point, ^1^H NMR, ^13^C NMR and ^31^P NMR. Then, it was used as a novel and capable catalyst for the synthesis of pyranopyrimidines from the multicomponent condensation reaction of barbituric acid, 4-hydroxycoumarin, and aromatic aldehydes in mild conditions, short reaction times, and high yields.

## Introduction

The attention of scientists and industrial interests has been attracted by the development of simple, efficient, green, and low-cost methods for the synthesis of organic compounds in this century. Although convention strategies are primarily focused on high-throughput methods in the shortest time, modern methods are eager to improve reusability, avoid waste generated, and minimize toxicity. To minimize the amount of toxic byproducts, hazardous reagents should be replaced with safer sources and greener and more environmentally friendly methods^1^. The concept of "green chemistry" refers to the means of harnessing natural resources to improve reaction efficiency, including the development and implementation of new chemical processes and transformations that are more efficient, safer, and more environmentally friendly^2,3^. Solvents have a strategic position thanks to the green chemistry framework. The solvent of a green environment should have sundry criteria such as availability, non-toxicity, recyclability, thermal stability, non-flammability, renewable, low vapor pressure, frugality, and bio-degradability^4,5^.

Deep eutectic solvents (DES) were first reported by the Abbott research group^6^. These compounds are a mixture of hydrogen bond acceptor groups (HBA) such as ammonium or phosphonium-based salts and hydrogen bond donor compounds (HBD) such as alcohols, amines and acids, which are synthesized under mild conditions^6,7^. DESs are an alternative class of ionic liquids (ILs) that have attracted the attention of many scientists in recent years^8–10^. The limitations of ILs are their toxicity, poor biodegradability, and high production cost, which DES have replaced as a new type of green solvent, and they do not have these limitations and are economically viable^11,12^. In addition, DES is easy to prepare and has many advantages such as high purity, low synthesis cost, high thermal stability, non-toxicity, reusability and bio-degradability. Moreover, they can be used as catalytically active species in many reactions^13^.

Therefore, the application and efficiency of DES as a green and environmentally friendly catalyst and or solvent in the synthesis of many compounds is of great importance and cannot be denied^14–17^. To date, various types of DES have been synthesized that offer significant advantages in organic synthesis.^1^

A multicomponent reaction (MCR) is a reaction in which three or more reagents are added simultaneously to a reaction flask and mixed in a one-pot process. These reactions have several advantages over linear synthesis protocols, including Fewer steps, and no need to isolate reaction intermediates (resulting in fewer purification steps), Since most of the carbon atoms are present in the final product, MCR can be considered as a very atom-economic process^18–20^.

*N*-heterocycles are organic compounds that contain at least one nitrogen atom in the ring, which often show unique properties and reactivities. These compounds have various applications in different fields such as pharmaceuticals, agricultural chemicals, and material science. In addition, they mainly have antibacterial, anti-tumor, anti-Alzheimer, anti-fungal, anti-inflammatory, anti-depressant, etc. properties. The appropriate methods for preparation of new heterocycles that can be used in various fields are the use of multi-component reactions, which increase the efficiency of these products and create appropriate economic benefits for the preparation of these compounds^21–31^. Among of *N*-heterocycles, pyranopyrimidine moiety widely exists in the structure of various natural products and has been shown to have various pharmacological activities such as antibronchitis^32^, antimicrobial^33^, anti-tumor^34,35^, antihyper-tensive, anticoagulant, antitumor, cardiac, liver protection, and anti-bronchitis activities^36–42^, and several methods reported for their syntheses^43–49^. Also, chromenes have many important biological properties such as anti-anaphylactic, anti-cancer, anti-coagulant, spasmolytic, diuretic, etc., and have received a lot of attention^50–54^. Therefore, the chromenopyrano[2,3-*d*]-pyrimidine system seems to be interesting because it has chromene and a pyranopyrimidine ring, both of which are promising for biological responses^55^. The synthesis of chromeno-pyranopyrimidines was first reported in 2014 by tetra-n-butylammonium tetrafluoroborate (TBAF) in CH_3_CN^56^.

**Scheme 1:**
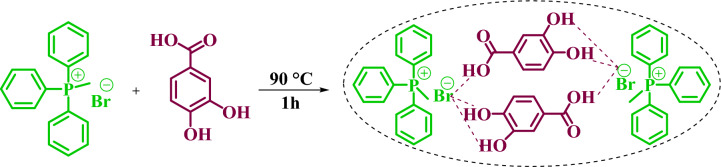
Synthesis of MTPPBr–PCAT–DES as a novel DES.

In this report, the aim is to design and synthesize MTPPBr–PCAT–DES as a novel DES for the synthesis of pyranopyrimidines that have potential biological properties. Compared with other DESs, the synthesized DES has advantages such as an easy synthesis process, low melting point, high purity, non-toxicity, biodegradability, and lower price. Since deep eutectic solvents have found many applications, a novel Deep Eutectic Solvent (MTPPBr–PCAT–DES) was prepared from a one-to-one mole ratio of MTPPBr and PCAT (Scheme 1) and characterized by various techniques such as FT-IR, TGA/DTA, densitometer, eutectic point, and ^1^H NMR.

**Scheme 2:**
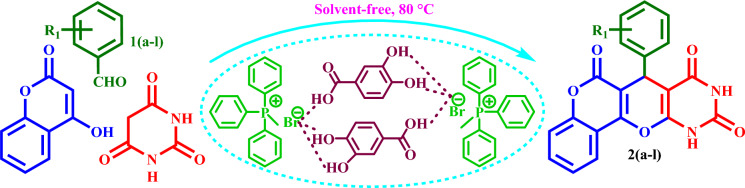
Synthesis of **2(a-l)** by MTPPBr–PCAT–DES.

Then, it was used as a capable and novel catalyst for the synthesis of seven new **2(a-f)** and five **2(g-l)** known chromenopyranopyrimidines from the reaction of barbituric acid, 4-hydroxy- coumarin, and aromatic aldehydes in solvent-free conditions at 80 °C (Scheme 2).

The key advantages of this new protocol are the use of inexpensive DES as a green solvent and catalyst, a one-step MCR reaction, good to excellent yields, short reaction times, mild reaction conditions, and reusability, which have several aspects of green and sustainable chemistry.

## Experimental

All reagents were purchased from the chemical companies Merck or Sigma Aldrich and used as received. Thin layer chromatography (TLC) was performed on silica gel 60 F-254 [Merck, Germany]. NMR was recorded on a Bruker DRX-250 using DMSO-*d*_*6*_ as solvent. FT-IR (KBr) spectra were recorded using an Alpha Perkin Elmer spectrophotometer. Melting points were determined using a Stuart melting point meter. Thermo-Gravimetric-Differential Thermal Analysis (TGA–DTA) was performed using the SDT Q600 V20.9 Build 20 device. The density of the DES catalyst was determined using AND-HR200.

### General procedure for preparation of MTPPBr–PCAT–DES

The mixture (molar ratio 1:1) of MTPPBr (1 mmol) and PCAT (1 mmol) was stirred at 90 °C in solvent-free conditions until a homogeneous liquid was obtained. When it cools slowly at room temperature, it turns into a transparent solid which dissolves well in water or ethanol.

### General procedure for the synthesis of** 2(a-l)**

MTPPBr–PCAT–DES (1.5 mmol), barbituric acid (1 mmol, 128 mg), 4-hydroxycoumarin (1 mmol, 174 mg), and aldehyde (1.0 mmol) were mixed in the presence of MTPPBr–PCAT–DES (1 mmol, 0.511 g) as a novel DES catalyst and stirred at 80 °C in solvent‐free condition for an appropriate time. After completion of the reaction (TLC), the resulting mixture was washed with ethanol and filtered to separate the catalyst from other materials (the reaction mixture is insoluble in ethanol and the catalyst is soluble). Upon completion of the reaction, a solid mass precipitated out and was filtered off, followed by the purification of the crude product just by washing it with cold aqueous ethanol.

The structure of each purified compound was confirmed with a comparison of their FT-IR, ^1^ HNMR, ^13^C NMR, and Mass spectra with authentic samples as well as their melting points.

## Results and discussion

### Synthesis of MTPPBr–PCAT

Deep eutectic solvents are an emerging class of ionic liquids that have expanded in recent years as environmentally friendly compounds that play both the role of a solvent and a catalyst. The catalytic performance of these compounds is based on the creation of hydrogen bonds, which increase their selectivity. It should be noted that the synthesis method of deep eutectic solvents is very easy and they are synthesized by mixing and heating without the need for purification and separation. Therefore, the mixture of MTPPBr and PCAT (molar ratio 1:1) was heated at 90 °C until a homogeneous liquid (DES) was obtained.

### Characterization of MTPPBr–PCAT–DES

The novel DES was characterized by FT-IR, TGA–DTA, densitometer, and eutectic points.

### Characterization by FT-IR

**Figure 1:**
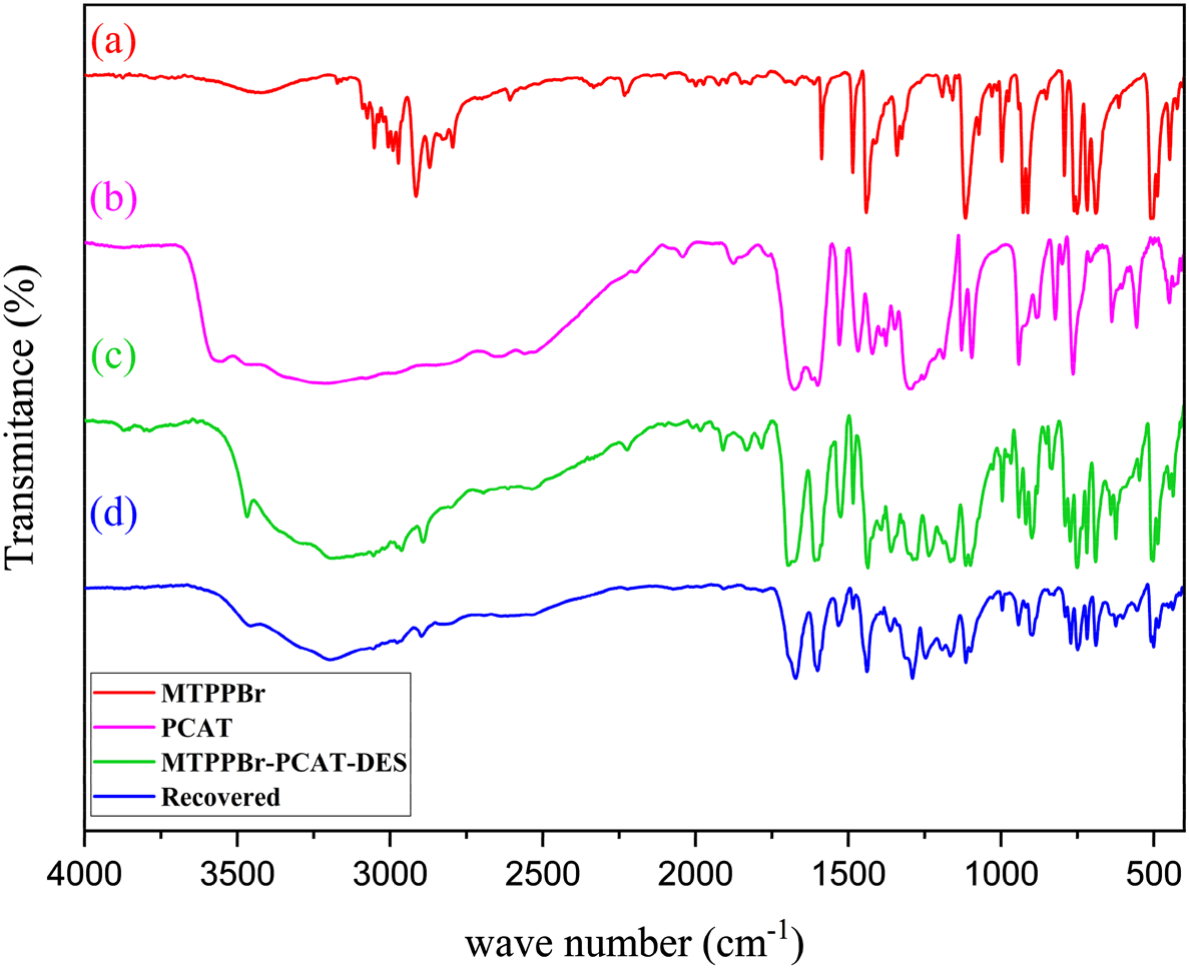
The FT-IR spectra of (**a**), (**b**), (**c**), and (**d**).

Figure 1 shows the FT-IR spectra of MTPPBr (a), PCAT (b), the fresh MTPPBr–PCAT–DES (DES catalyst) (c), and the recovered MTPPBr–PCAT–DES (DES catalyst) (d).

In spectrum (a), the peaks at about 2900–3100 cm^−1^ belong to the aromatic and aliphatic hydrogens, and the peaks at about 750 and 1480 cm^−1^ are related to the C-P bonds, respectively. In spectrum (b), the peaks at 3226 and 1675 cm^−1^ are related to the O–H and C=O of the –COOH group, respectively. The (c) spectrum shows the similar peaks shown in both (a) and (b) spectra confirming the structure of the DES catalyst. The peaks related to the O–H and C=O groups appeared with slight shifts at 3183 and 1697 cm^−1^, indicating the successful DES synthesis.

There is a nice similarity between the FT-IR spectra of (c), and (a) and (b) with slight shifts in the related peaks. As we know, when the hydrogen bond forms, the bond vibration requirs less energy, the vibration is easier and is transferred to a lower frequency (more wavelength) as can be seen in the FT-IR spectrum of (c) compared to the FT-IR spectra of (a) and (b).

### Characterization by ^1^H NMR

#### The ^1^H NMR spectrum of MTPPBr

In addition to other methods, the ^1^H NMR, ^13^C NMR, and ^31^P NMR techniques were also used for further confirmation.

**Figure 2:**
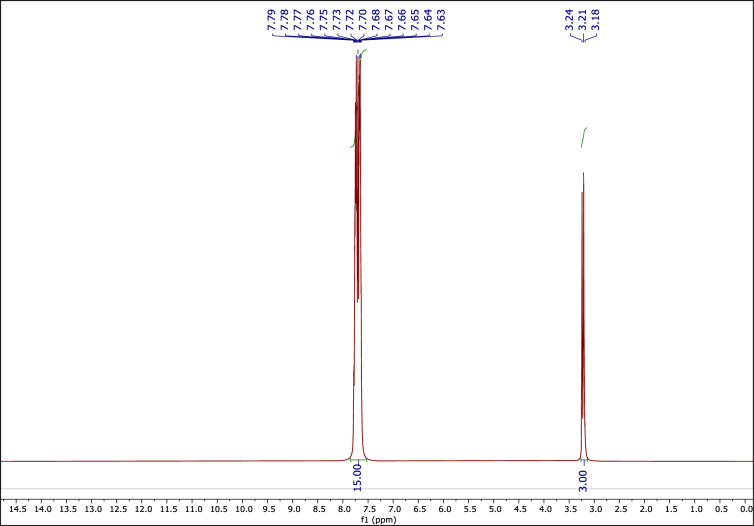
The ^1^H NMR of MTPPBr.

Figure 2 shows the ^1^H NMR spectrum of MTPPBr. The peaks at 3.23 (d, 3H), and 7.63–7.79 (m, 15H) ppm are related to the CH_3_ hydrogens, and the three phenyl rings, respectively.

#### The ^1^H NMR spectrum of PCAT

**Figure 3:**
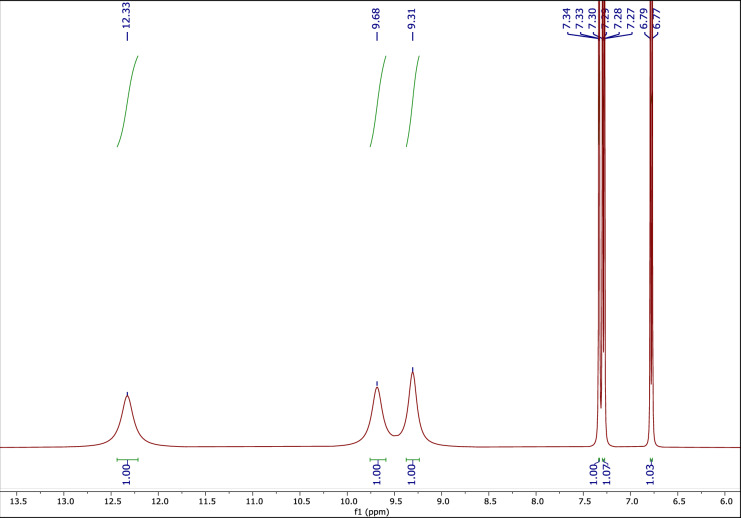
The ^1^H NMR of PCAT.

Figure 3 shows the ^1^H NMR spectrum of PCAT. The peaks at 6.77–7.34 (3H, aromatic), 9.31 (s, 1H), 9.68 (s, 1H), and 12.33 (s, 1H), ppm are related to the phenyl ring, the two OH groups and the OH of the acid group, respectively.

#### The ^1^H NMR spectrum of the DES catalyst

**Figure 4:**
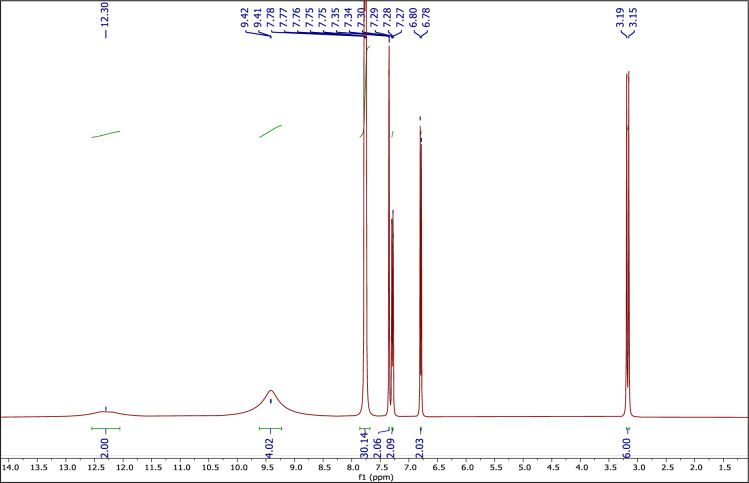
The ^1^H NMR of MTPPBr–PCAT–DES.

Figure 4 shows the ^1^H NMR spectrum of DES compound. The peaks at 3.15–3.19 (m, 6H) ppm are related to the two CH_3_ hydrogens, the peaks at 6.78–7.35 (m, 6H) ppm are related to the two phenyl ring of PCAT, the peaks at 7.75–7.90 (m, 30H) ppm are related to the six phenyl rings of MTPPBr, the peaks at 9.41–9.42 (s, 4H) are related to the four hydroxy of PCAT and the peak at 12.30 ppm (s, 2H) are related to the two hydroxy of acid groups. When DES is formed, the signal intensity of the active hydrogens weakens and shifts towards the low field. These observations indicate the hydrogen bond interactions between MTPPBr and PCAT,^57^ confirming the structure of the newly formed DES.

#### The ^13^C NMR spectrum of the DES catalyst

**Figure 5:**
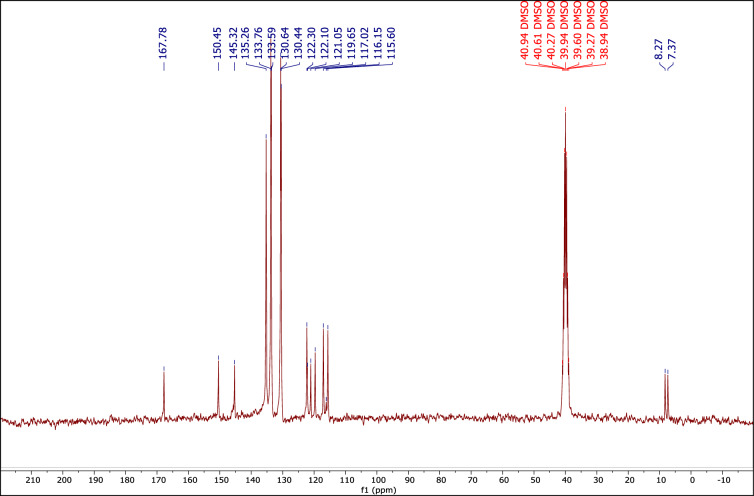
The ^13^C NMR of MTPPBr–PCAT–DES.

Figure 5 shows the ^13^C NMR spectrum of MTPPBr–PCAT–DES with seventeen peaks at 167.7, 150.4, 145.3, 135.2, 133.7, 133.5, 130.6, 130.4, 122.3, 122.1, 121, 191.6, 117, 116, 115.6, 8.27 and 7.37 ppm, which confirm the DES structure.

#### The ^31^P NMR spectrum of the DES catalyst

**Figure 6:**
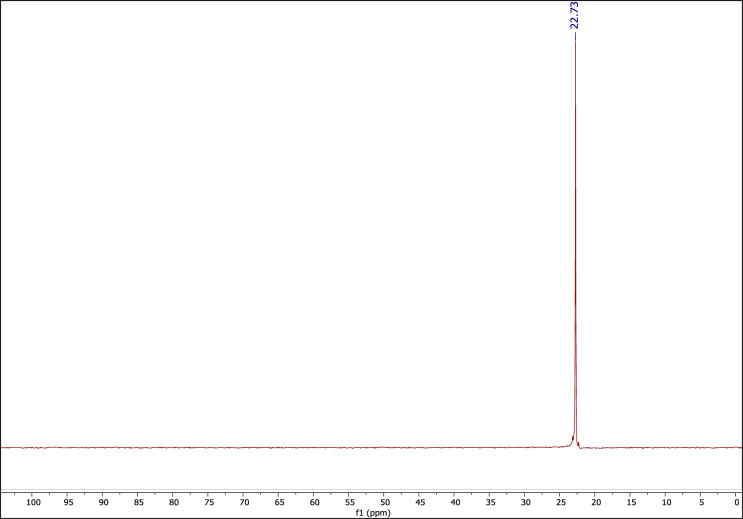
The ^31^P NMR of MTPPBr–PCAT–DES.

Figure 6 shows the ^31^P NMR spectrum of the DES catalyst which confirms the presence of the phosphorous in the structure of the molecule and its formation^58^.

### Characterization by TGA–DTA

**Figure 7:**
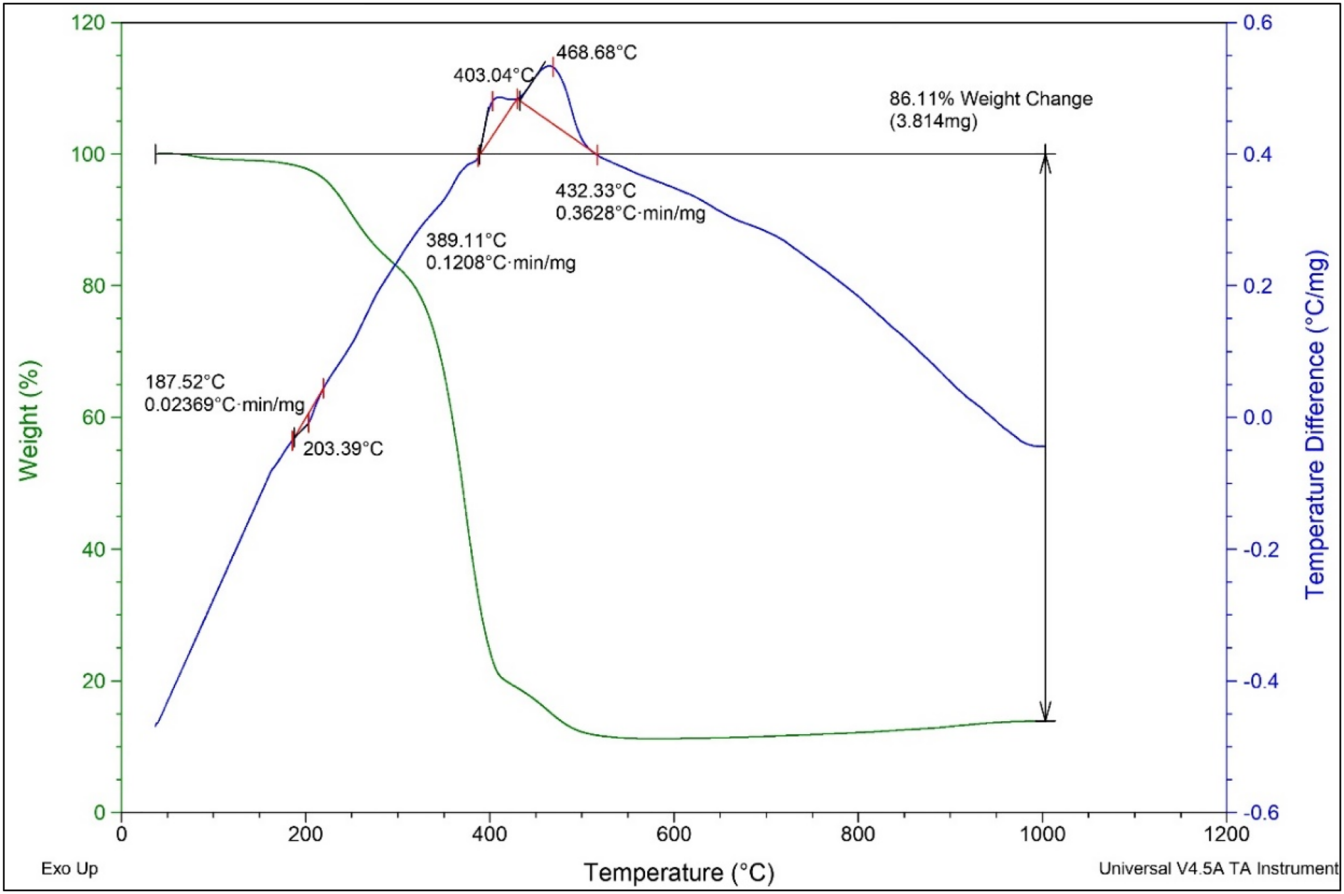
The TGA–DTA curve of MTPPBr–PCAT.

To investigate the stability and thermal behavior of MTPPBr–PCAT–DES, the TGA–DTA analysis was performed (Fig. 7). The curve has two main breaks in the 389 °C and 432 °C regions. The first weight loss at about 389 °C is related to the removal of the acidic compound and the breakdown of the hydrogen bond. The last weight loss at about 432 °C is related to the breakdown of the ionic bonds.

However, according to the DTG, there are two small breaks in the area below 200 °C, which can be related to the very small amount of water released during the preparation of the catalyst, and can also be related to the noise of the device. If it is related to the release of water, according to the graph, this release of water is very little and no change has been made in the structure, and these results are inferred from the conducted ^1^HNMR analysis and the structure of the catalyst has not been damaged. These results indicate the high thermal stability of MTPPBr–PCAT.

### Characterization by densitometer

Density is the thermophysical property most studied for DESs, and most of them are considered to have a density in the range of 1.0 and 1.35 g/cm^3^ at 298.15 K, which is higher than the density of water. So, a sample with a certain weight is mixed with a certain volume of water, then by using the formula (ρ = P_w_/1 − m_w_/m_d_), the density is calculated. The density of MTPPBr–PCAT–DES is about 1.2409 g/mL (Table 1)^59^.

**Table 1:** The MTPPBr–PCAT–DES catalyst density data.

The mass of 1.0 mL of water (g, P_w_)	Wet sample mass (g, M_w_)	Sample dry mass (g, M_d_)
0.99618	0.0368	0.1868
ρ = P_w_/1 − m_w_/m_d_ = 0.99618/1–0.0368/0.1868 = 1.2409 g/mL		

### Characterization by Eutectic Points

**Figure 8:**
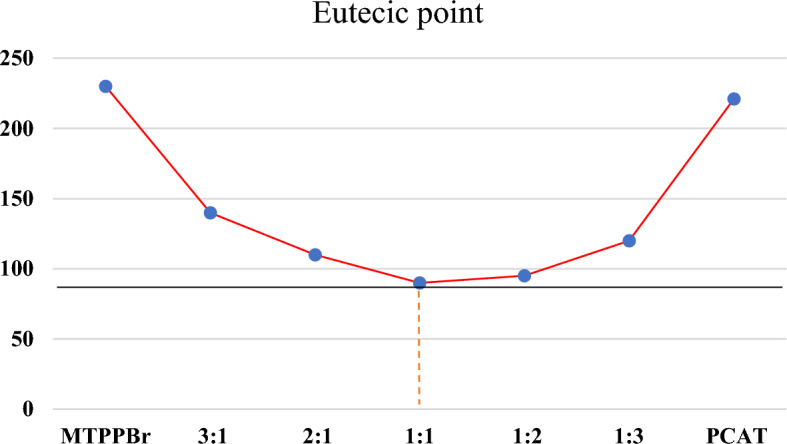
The eutectic points phase diagram of MTPPBr–PCAT–DES.

To check the best ratio of MTPPBr to PCAT, the eutectic point experiment was performed, and different ratios of MTPPBr to PCAT were prepared. The eutectic point phase diagram (Fig. 8) showed that the best ratio for the novel DES formation is one mole of MTPPBr to one mole of PCAT. The melting points of MTPPBr and PCAT are 230 and 221 °C, respectively, but when a novel DES was prepared, the melting point decreased to 90 °C.

### Optimization of the reaction conditions for the synthesis of 2 h

**Scheme 3:**
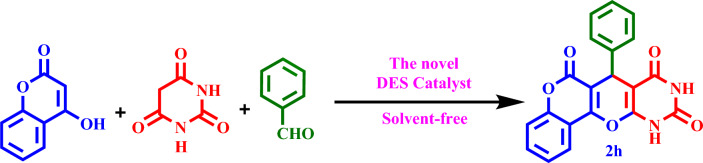
Synthesis of **2h** by the novel DES catalyst.

To optimize the preparation conditions of the target products, the reaction between barbituric acid, 4‐hydroxycoumarin, and benzaldehyde (synthesis of **2 h**) was selected as a model reaction (Scheme 3). This reaction was evaluated using different solvents and the solvent-free condition, which the solvent-free condition was chosen as the suitable condition.

Also, performing the model reaction was checked at different temperatures, and 80 °C was chosen as an appropriate temperature. Then, different amounts of the catalyst were used to find the optimum amount of the catalyst.

The best result was found to be the 1:1:1 mol ratio of barbituric acid, 4‐hydroxycoumarin, and benzaldehyde with 1.5 mmol of the novel DES catalyst at 80 °C in solvent-free conditions (Table 2).

**Table 2:** Optimization of the reaction conditions in the synthesis of **2h** compounds.

Entry	Catalyst (mmol)	Temp. (°C)	Solvent	Yield (%)
1	0.5	80	–	87
2	1.0	80	–	90
3	2.0	80	–	93
4	2.5	80	–	92
5	3.0	80	–	89
6	1.5	60	–	85
7	1.5	70		88
8	1.5	90	–	91
9	1.5	100	–	90
10	1.5	110	–	82
11	1.5	Reflux	EtOH	88
12	1.5	Reflux	H_2_O/EtOH	71
13	1.5	Reflux	H_2_O	50
14	1.5	Reflux	*n*-Hexane	55
15	1.5	Reflux	EtOAc	78
**16**	**1.5**	**80**	**–**	**95**

### Synthesis of 2(a-j)

Based on the obtained results from the model reaction (synthesis of **2h**), **2(a-l)** were synthesized from the reaction of barbituric acid, 4-hydroxycoumarin and aldehyde with 1.5 mmol of the DES catalyst at 80 °C in solvent-free conditions (Table 3). Short reaction times and high yields are the important features of this proposed method.

**Table 3:** Synthesis of **2(a-l)** by MTPPBr–PCAT–DES.

Entry	Aldehyde	Product	Time (min.)	Yield (%)	M.P. °C Found, literature	TON	TOF
1		**2a**	25	85	255–258, NEW	56.66	2.26
2		**2b**	20	96	306–310, NEW	64.00	3.20
3		**2c**	15	94	250–253, NEW	62.66	4.17
4		**2d**	20	91	242–246, NEW	60.66	3.03
5		**2e**	20	88	219–222, NEW	58.66	2.93
6		**2f**	40	95	295–298, NEW	63.33	1.58
7		**2g**	30	90	350 > 60	60.00	2.00
8		**2h**	10	95	275–278, 300 > 55	63.33	6.33
9		**2i**	15	91	256–259, 300 > 55	60.66	4.02
10		**2j**	20	87	248–250, 60	58.00	2.90
11		**2k**	15	92	290–293, 300 > 55	61.33	4.08
12		**2l**	15	90	280–282, 300 > 55	60.00	4.00

### Spectral data

#### 7-(2,4-Dichlorophenyl)-7,11-dihydro-6*H*,8*H*-chromeno[3',4':5,6]pyrano[2,3-*d*]pyrimidine-6, 8,10(9*H*)-trione (**2a**)

Light Yellow solid, M.P.: 255–258 °C; IR (KBr) ν = 3406, 3208, 3074, 2854, 1760, 1723, 1690, 1578, 1469, 1439, 1380, 1326, 1226, 1110, 791, 543 and 437 cm^-1^. ^1^H NMR (250 MHz, DMSO-*d*_*6*_) δ = 11.49 (s, 1H), 11.28 (s, 1H), 8.32–7.09 (m, 7H), 4.55 (s, 1H). ^13^C NMR (62.5 MHz, DMSO-*d*_*6*_) δ = 162.9, 161.3, 150.6, 148.8, 135.9, 134.5, 133.5, 131.8, 128.8, 127.0, 124.0, 122.7, 121.1, 116.2, 113.4, 105.3, 103.3, 100.6, 82.9, 31.7.; MS: m/z = 429.2 [M]^+^, base peak: m/z = 120.1.
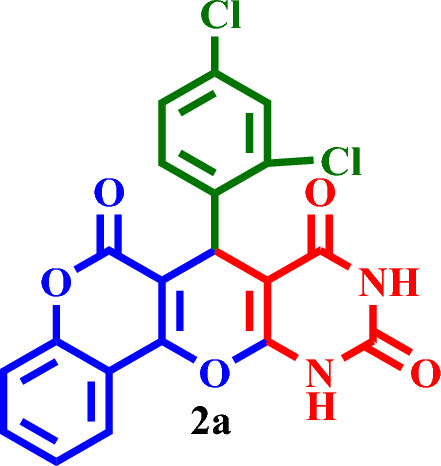


#### 7-(3,4-Dimethoxyphenyl)-7,11-dihydro-6*H*,8*H*-chromeno[3',4':5,6]pyrano[2,3-*d*]pyrimidine-6,8,10(9*H*)-trione (**2b**)

Yellow solid, M.P.: 306–310 °C; IR (KBr) ν = 3224, 3142, 3073, 2991, 2839, 1740, 1696, 1541, 1655,1541,1496, 1390, 969, 767 and 524 cm^-1^. ^1^H NMR (250 MHz, DMSO-*d*_*6*_) δ = 11.28 (s, 1H), 11.16 (s, 1H), 7.87 (d, *J* = 8.1 Hz, 1H), 7.56 (t, *J* = 7.7 Hz, 1H), 7.36–7.29 (m, 2H), 7.08 (d, *J* = 8.5 Hz, 1H), 6.80–6.63 (m, 2H), 6.25 (s, 1H), 3.88–3.84 (m, 3H), 3.79 (s, 3H).^13^C NMR (62.5 MHz, DMSO-*d*_*6*_) δ = 165.8, 165.1, 164.4, 162.8, 155.9, 152.6, 150.6, 148.2, 132.1, 125.7, 124.3, 124.0, 119.3, 118.6, 117.2, 116.3, 111.5, 104.7, 83.8, 56.2, 55.9, 36.0.; MS: m/z = 420.3 [M]^+^, base peak: m/z = 276.1.
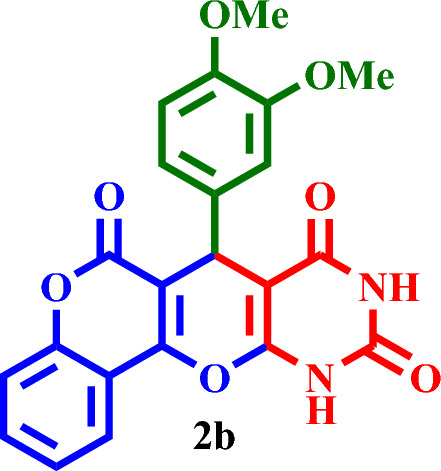


#### 7-(4-(Dimethylamino)phenyl)-7,11-dihydro-6*H*,8*H*-chromeno[3',4':5,6]pyrano[2,3-*d*]pyrimi- dine-6,8,10(9*H*)-trione (**2c**)

Red solid, M.P.: 250–253 °C; IR (KBr) ν = 3549, 3423, 3171, 3060, 2831, 1660, 1606, 1514, 1405, 1377, 1310, 1210, 944, 790, 529, 511 and 408 cm^−1^. ^1^H NMR (250 MHz, DMSO-*d*_*6*_) δ = 11.02 (s, 1H), 10.89 (s, 1H), 7.79 (d, *J* = 7.5 Hz, 1H), 7.49 (t, *J* = 7.6 Hz, 2H), 7.35 (d, *J* = 8.2 Hz, 1H), 7.24–7.18 (m, 4H), 6.26 (s, 1H), 3.44 (s, 6H). ^13^C NMR (62.5 MHz, DMSO-*d*_*6*_) δ = 168.0, 165.1, 163.1, 155.9, 154.6, 152.9, 150.7, 139.4, 131.5, 128.6, 124.5, 123.4, 120.4, 119.6, 116.0, 111.6, 109.9, 103.5, 91.6, 45.7, 40.0, 36.3.; MS: m/z = 403.3 [M]^+^, base peak: m/z = 259.1.
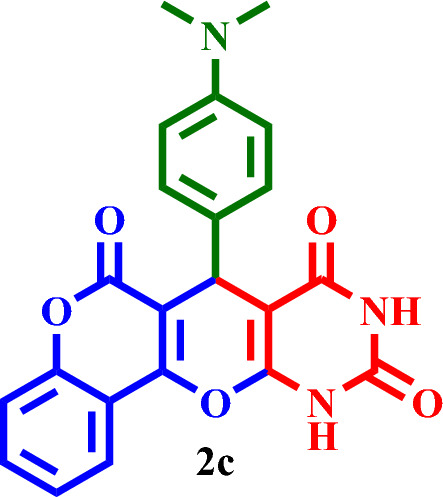


#### 7-(4-(Diethylamino)phenyl)-7,11-dihydro-6*H*,8*H*-chromeno[3',4':5,6]pyrano[2,3-*d*]pyrimi-dine-6,8,10(9*H*)-trione (**2d**)

Crimson solid, M.P.: 242–246 °C; IR (KBr) ν = 3427, 3195, 3068, 2977, 1726, 1673, 1614, 1502, 1450, 1392, 1189, 1154, 792 and 514 cm^-1^. ^1^H NMR (250 MHz, DMSO-*d*_*6*_) δ = 10.98 (d, *J* = 11.6 Hz, 1H), 10.85 (d, *J* = 11.6 Hz, 1H), 9.03–5.78 (m, 9H), 3.48 (q, *J* = 6.9 Hz, 4H), 1.11 (q, *J* = 6.6 Hz, 6H). ^13^C NMR (62.5 MHz, DMSO-*d*_*6*_) δ = 168.1, 165.2, 163.1, 155.7, 152.6, 150.7, 139.9, 133.1, 131.5, 128.8, 124.3, 123.6, 121.9, 120.1, 116.7, 115.9, 111.3, 109.4, 105.1, 91.4, 44.6, 34.0, 12.9, 10.7.; MS: m/z = 431.6 [M]^+^, base peak: m/z = 272.1.
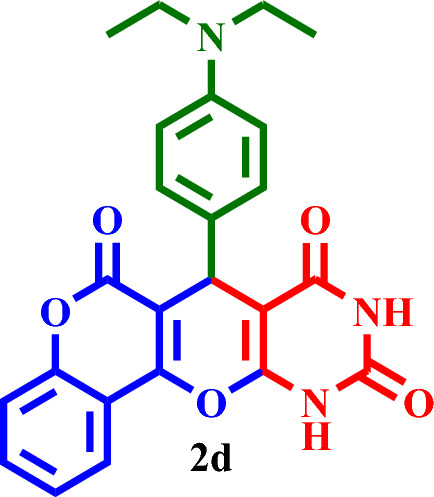


#### 7-(Furan-2-yl)-7,11-dihydro-6*H*,8*H*-chromeno[3',4':5,6]pyrano[2,3-*d*]pyrimidine-6,8,10(9*H*)- trione (**2e**)

Yellow solid, M.P.: 219–222 °C; IR (KBr) ν = 3447, 3200, 3149, 3045, 2852, 1743, 1703, 1655, 1563, 1432, 1404, 1362, 1317, 1029, 930, 572, 517 and 410 cm^-1^. ^1^H NMR (250 MHz, DMSO-*d*_*6*_) δ = 11.32 (s, 1H), 11.23 (s, 1H), 7.87 (d, *J* = 7.7 Hz, 1H), 7.56 (d, *J* = 7.8 Hz, 1H), 7.30 (d, *J* = 8.6 Hz, 2H), 6.88 (s, 2H), 6.25 (d, *J* = 10.5 Hz, 1H), 6.00 (s, 1H).^13^C NMR (62.5 MHz, DMSO-*d*_*6*_) δ = 166.2, 164.6, 163.7, 162.5, 152.6, 151.6, 150.6, 141.6, 137.3, 132.2, 126.9, 124.4, 115.6, 113.2, 110.6, 106.2, 91.4, 31.9.; MS: m/z = 350.3 [M]^+^, base peak: m/z = 206.1.
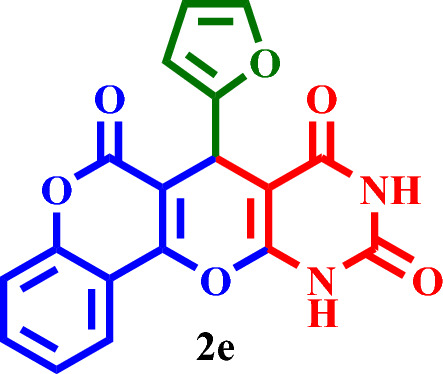


#### 4-(6,8,10-Trioxo-8,9,10,11-tetrahydro-6*H*,7*H*-chromeno[3',4':5,6]pyrano[2,3-*d*]pyrimidin-7-yl)benzoic acid (**2f**):

White solid, M.P.: 295–298 °C; IR (KBr) ν = 3271, 3246, 3134, 2935, 2874, 1766, 1735, 1704, 1599, 1467, 1417, 1366, 1219, 1289, 1042, 965, 744, 538, 502 and 452 cm^−1^. ^1^H NMR (250 MHz, DMSO-*d*_*6*_) δ = 11.57 (s, 1H), 11.33 (s, 1H), 10.95 (s, 1H), 7.83–7.52 (m, 8H), 6.23 (s, 1H). ^13^C NMR (62.5 MHz, DMSO-*d*_*6*_) δ = 191.3, 169.6, 166.2, 162.8, 162.7, 151.6, 150.7, 148.2, 134.6, 134.6, 133.3, 129.4, 127.4, 125.7, 125.6, 123.2, 123.2, 83.4, 78.9, 51.1; MS: m/z = 403.2 [M]^+^, base peak: m/z = 231.1.
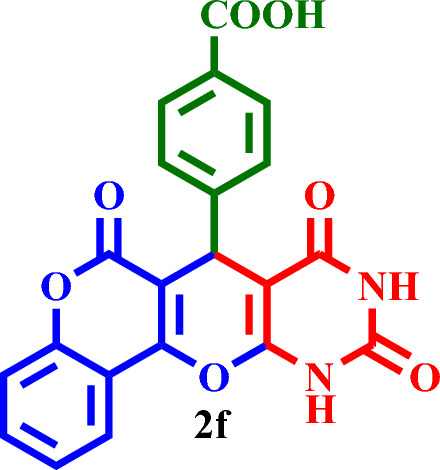


#### 4-(6,8,10-Trioxo-8,9,10,11-tetrahydro-6*H*,7*H*-chromeno[3',4':5,6]pyrano[2,3-*d*]-pyrimidin-7-yl)benzaldehyde (**2g**)

Light Yellow solid, M.P.: 350 > °C; IR (KBr) ν = 3422, 3206, 3093, 2943, 2841, 1743, 1606, 1573, 1439, 1406, 1306, 1209, 1025, 762 and 540 cm^-1^. ^1^H NMR (250 MHz, DMSO-*d*_*6*_) δ = 11.42 (s, 1H), 11.27 (s, 1H), 9.90 (s, 1H), 7.83 (d, *J* = 8.3 Hz, 2H), 7.73 (d, *J* = 7.8 Hz, 1H), 7.55 (d, *J* = 7.5 Hz, 1H), 7.32 (t, *J* = 7.9 Hz, 4H), 6.35 (s, 1H).
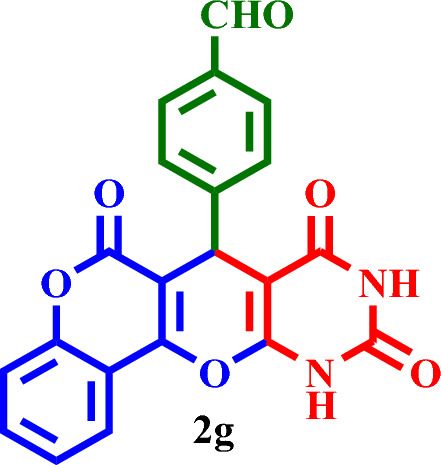


#### 7-Phenyl-7,11-dihydro-6*H*,8*H*-chromeno[3',4':5,6]pyrano[2,3-*d*]pyrimidine-6,8,10(9*H*)-trione (**2h**)

White solid, M.P.: 275–278 °C; IR (KBr) ν = 3400, 3213, 3059, 1727, 1655, 1621, 1494, 1450, 1296, 1195, 1031, 953, 798, 524 and 460 cm^-1^. ^1^H NMR (250 MHz, DMSO-*d*_*6*_) δ = 11.29 (d, *J* = 39.3 Hz, 2H), 8.28–7.09 (m, 9H), 6.02 (s, 1H).
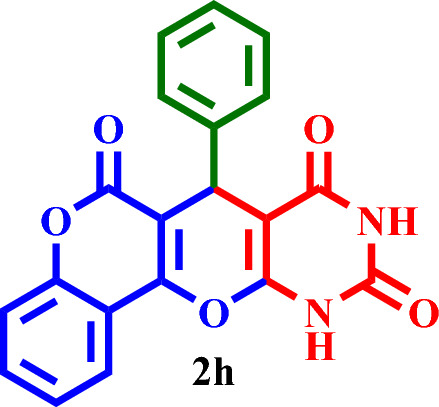


#### 7-(4-Chlorophenyl)-7,11-dihydro-6*H*,8*H*-chromeno[3',4':5,6]pyrano[2,3-*d*]-pyrimidine-6,8, 10(9*H*)-trione (**2i**)

White solid, M.P.: 256–259 °C; IR (KBr) ν = 3429, 3075, 2989, 2612, 1670, 1603, 1562, 1490, 1352, 1311, 1094, 907, 765 and 498 cm^−1^.
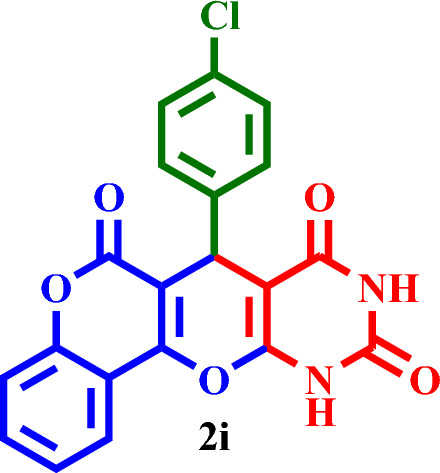


#### 7-(*p*-Tolyl)-7,11-dihydro-6*H*,8*H*-chromeno[3',4':5,6]pyrano[2,3-*d*]pyrimidine-6,8,10(9*H*)-trione (**2j**)

White solid, M.P.: 248–250 °C; IR (KBr) ν = 3427, 3208, 3086, 2844, 1752, 1701, 1672, 1443, 1573, 1296, 1219, 1192, 1095, 795, 763, 636, 567 and 423 cm^−1^.
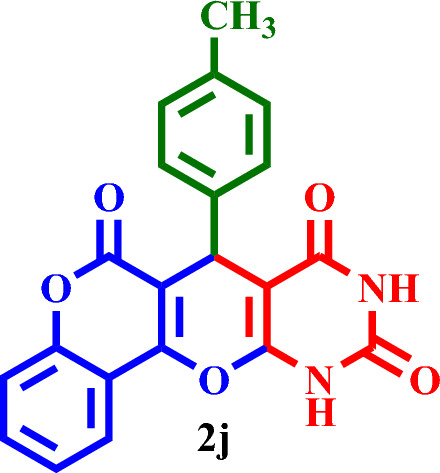


#### 7-(3-Nitrophenyl)-7,11-dihydro-6*H*,8*H*-chromeno[3',4':5,6]pyrano[2,3-*d*]pyrimidine-6,8,10 (9*H*)-trione (**2k**)

Yellow solid, M.P.: 290–293 °C; IR (KBr) ν = 3406, 2945, 2732, 1655, 1618, 1529, 1494, 1347, 1312, 1186, 1101, 913, 793, 761, 720, 676 and 463 cm^−1^.
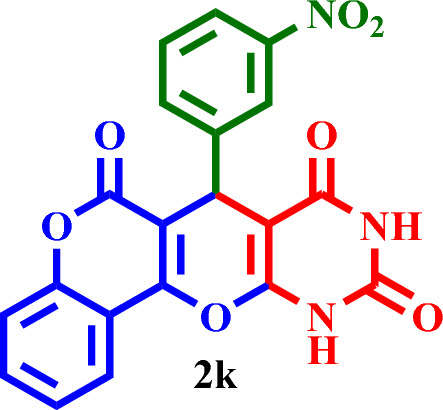


#### 7-(2-Chlorophenyl)-7,11-dihydro-6*H*,8*H*-chromeno[3',4':5,6]pyrano[2,3-*d*]pyrimidine-6,8,10- (9*H*)-trione (**2l**)

Yellow solid, M.P.: 290–293 °C; IR (KBr) ν = 3433, 3197, 3057, 2964, 1686, 1612, 1568, 1402 and 1188 cm^−1^.
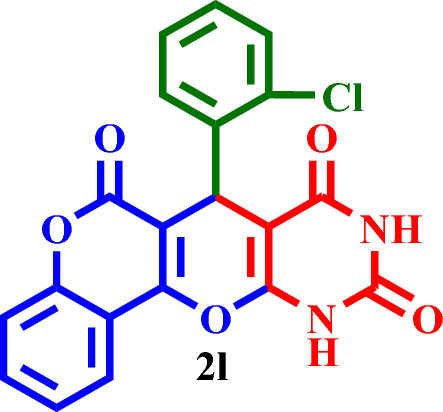


### A proposed mechanism for the synthesis of 2(a-l)

**Scheme 4:**
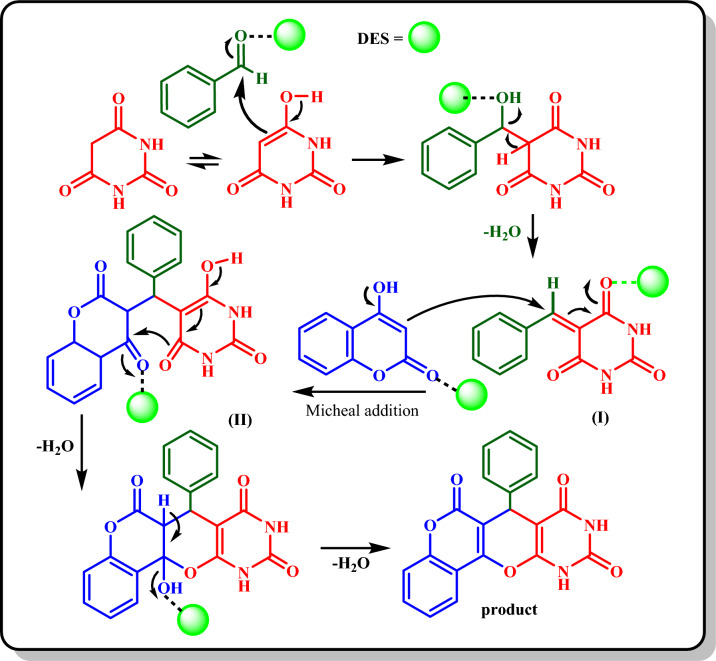
Proposed mechanism for the synthesis of **2(a-l).**

The possible mechanism for the synthesis of **2(a-l)** is shown in Scheme 4. First, barbituric acid is converted into its enol form by the novel DES catalyst and reacts with the DES-activated aldehyde with the deletion of an H_2_O molecule to form the intermediate (I). Then, intermediate (I) reacts as a Michael acceptor with 4‐hydroxycoumarin to form the intermediate (II). An intramolecular cyclization and deletion of a water molecule will change the intermediate (II) into the final product^59^.

### Reusability of the MTPPBr–PCAT–DES

**Figure 9:**
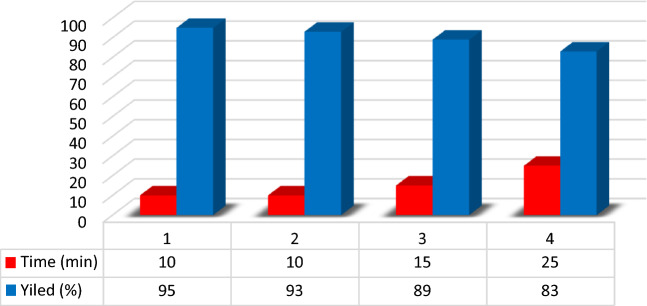
Reusability of the MTPPBr–PCAT–DES catalyst in four consecutive reaction runs.

To prove the efficiency of the novel DES catalyst, its recyclability was tested in four consecutive runs. Therefore, after completion of the model reaction, ethanol and water were added, and the reaction mixture filtered to separate the catalyst. The DES catalyst was separated from the filtrate by removing the ethanol and water solvents and used in next three successive runs with the similar reaction conditions. The results show relatively no significant loss of activity (95%, 93%, 89%, and 83%, respectively) (Table 4, Fig. 9).

**Table 4:** Amount of reused DES (g).

Fresh (Run 1)	Run-2	Run-3	Run-4
0.511 (g)	0.495 (g)	0.480 (g)	0.470 (g)

**Figure 10:**
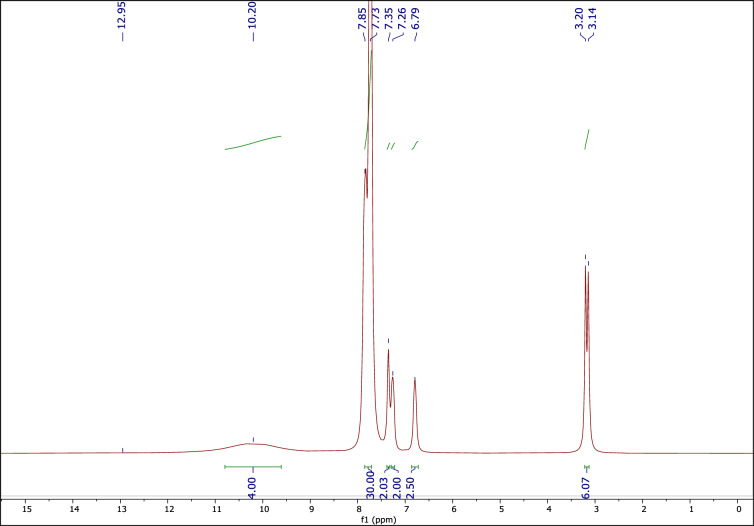
The ^1^H NMR of recovered catalyst.

**Figure 11:**
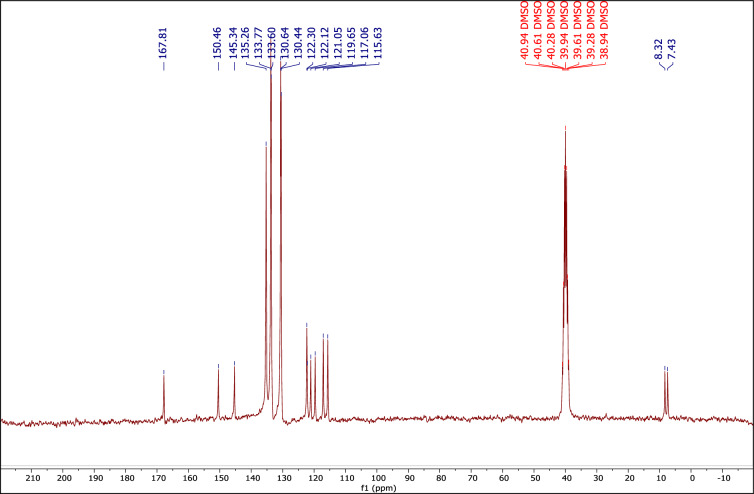
The ^13^C NMR of recovered catalyst.

**Figure 12:**
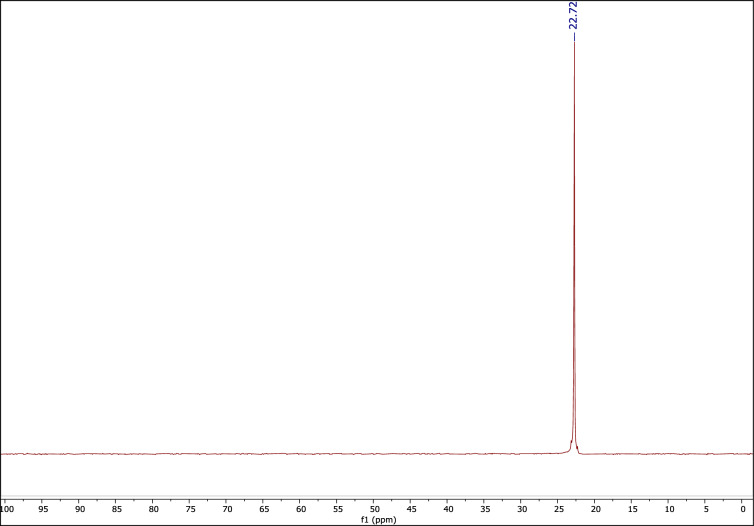
The ^31^P NMR of recovered catalyst.

Also, the ^1^H NMR, ^13^C NMP, and ^31^P NMR spectra of the used DES are very similar to the corresponding fresh one, indicating preservation of the DES structure during the reactions (Figs. 10, 11, 12).

### Comparison of the catalyst activities

Table 5 shows the comparison of different methods for the synthesis of **2(a-l)** with our proposed procedure.

**Table 5:** Comparison of the MTPPBr–PCAT–DES catalyst with the other catalysts.

Entry	Catalyst	Condition	Time (min)	Yield (%)	References
1	Trisodium citrate	EtOH:H_2_O (1:1 v/v)	35–60	74–95	^60^
2	Electrocatalytic	PrOH, TBAF	10–45	54–92	^55^
3	MTPPBr/PCAT–DES	Solvent-free, 80 °C	10–40	80–96	Our work

For example, **2h** was synthesized by trisodium citrate in EtOH:H_2_O (1:1 v/v) with 83% yield in 45 min^60^. Also, it was synthesized by an electrocatalytic method and tetra-n-butyl-ammonium fluoride (TBAF) in PrOH with 54% yield in 45 min^55^. This comparison reveals the advantage of our proposed method with performing the reaction in shorter reaction time and higher yields.

## Conclusion

In summary, a novel deep eutectic solvent (MTPPBr–PCAT–DES) was prepared from one mole of MTPPBr and one mole of PCAT (according to the eutectic point phase diagram) to expand the field of green and environmentally friendly catalysts, and the structure of the novel DES compound was confirmed by various techniques. Synthesis of deep eutectic mixtures is more energy efficient and can be synthesized simply by mixing and heating the components without purification. DESs can be effective not only as moderate, cheap, and environmentally safe solvents but also as recoverable and reusable organic catalysts to facilitate organic transformations.

Then, the novel DES was used as an efficient, powerful, and recyclable catalyst in the synthesis of pyranopyrimidines with short reaction times and high yields.

## Data and materials availability

All data generated or analyzed during this study are included in this published article and its Supplementary Information files.

### Supplementary Information

Supplementary Figures.

## References

[1] Dindarloo Inaloo I, Majnooni S, Esmaeilpour M (2018). Superparamagnetic Fe_3_O_4_ nanoparticles in a deep eutectic solvent: An efficient and recyclable catalytic system for the synthesis of primary carbamates and monosubstituted ureas. Eur. J. Org. Chem..

[2] Anastas P, Eghbali N (2010). Green chemistry: Principles and practice. Chem. Soc. Rev..

[3] Poliakoff M, Fitzpatrick JM, Farren TR, Anastas PT (2002). Green chemistry: Science and politics of change. Science.

[4] Constable DJ, Curzons AD, Cunningham VL (2002). Metrics to ‘green chemistry which are the best?. Green Chem..

[5] Newman SG, Jensen KF (2013). The role of flow in green chemistry and engineering. Green Chem..

[6] Abbott, A. P., Capper, G., Davies, D. L., Munro, H. L., Rasheed, R. K. & Tambyrajah, V. Preparation of novel, moisture-stable, Lewis-acidic ionic liquids containing quaternary ammonium salts with functional side chains electronic supplementary information (ESI) available: plot of conductivity vs. temperature for the ionic liquid formed from zinc chloride and choline chloride (2∶1). *Chem. Commun.* 2010–2011 (2001). 10.1039/b106357j 12240264

[7] Marset X, Pérez JM, Ramón DJ (2016). Cross-dehydrogenative coupling reaction using copper oxide impregnated on magnetite in deep eutectic solvents. Green Chem..

[8] Zhang Q, Vigier KDO, Royer S, Jérôme F (2012). Deep eutectic solvents: Syntheses, properties and applications. Chem Soc Rev..

[9] Dai Y, Van Spronsen J, Witkamp GJ, Verpoorte R, Choi YH (2013). Ionic liquids and deep eutectic solvents in natural products research: Mixtures of solids as extraction solvents. J. Nat. Prod..

[10] Dindarloo Inaloo I, Majnooni S (2019). Carbon dioxide utilization in the efficient synthesis of carbamates by deep eutectic solvents (DES) as green and attractive solvent/catalyst systems. New J. Chem..

[11] Alavi L, Seidi S, Jabbari A, Baheri T (2017). Deep eutectic liquid organic salt as a new solvent for carrier-mediated hollow fiber liquid phase microextraction of lead from whole blood followed by electrothermal atomic absorption spectrometry. New J. Chem..

[12] Dindarloo Inaloo I, Majnooni S (2019). Deep eutectic solvents (des) as green and efficient solvent/catalyst systems for the synthesis of carbamates and ureas from carbonates. ChemistrySelect.

[13] Bagherzadeh N, Sardarian AR, Dindarloo Inaloo I (2021). Green and efficient synthesis of thioureas, ureas, primary O-thiocarbamates, and carbamates in deep eutectic solvent/catalyst systems using thiourea and urea. New J Chem..

[14] Lindberg D, de la Fuente Revenga M, Widersten M (2010). Deep eutectic solvents (DESs) are viable cosolvents for enzyme-catalyzed epoxide hydrolysis. J. Biotechnol..

[15] Wagle DV, Zhao H, Baker GA (2014). Deep eutectic solvents: Sustainable media for nanoscale and functional materials. Acc. Chem. Res..

[16] Nguyen HT, Chau DKN, Tran PH (2017). A green and efficient method for the synthesis of pyrroles using a deep eutectic solvent ([CholineCl][ZnCl_2_]_3_) under solvent-free sonication. New J Chem..

[17] Alonso DA, Baeza A, Chinchilla R, Guillena G, Pastor IM, Ramón DJ (2016). Deep eutectic solvents: The organic reaction medium of the century. EurJOC..

[18] Batalha PN (2012). Recentes Avanços em Reações Multicomponentes: Uma Perspectiva entre os anos de 2008 e 2011. Rev. Virtual Quim..

[19] Horvath IT, Anastas PT (2007). Innovations and green chemistry. Chem. Rev..

[20] Biggs-Houck JE, Younai A, Shaw JT (2010). Recent advances in multicomponent reactions for diversity-oriented synthesis. Curr. Opin. Chem..

[21] Rezayati S, Kalantari F, Ramazani A, Sajjadifar S, Aghahosseini H, Rezaei A (2021). Magnetic silica-coated picolylamine copper complex [Fe_3_O_4_@SiO_2_@gp/picolylamine–Cu(ii)]-catalyzed biginelli annulation reaction. Inorg. Chem..

[22] Rezayati S, Ramazani A, Sajjadifar S, Aghahosseini H, Rezaei A (2021). Design of a Schiff base complex of copper coated on epoxy-modified core–shell MNPs as an environmentally friendly and novel catalyst for the one-pot synthesis of various chromene-annulated heterocycles. ACS Omega..

[23] Hosseinzadeh Z, Ramazani A, Razzaghi-Asl N (2018). Anticancer nitrogen-containing heterocyclic compounds. Curr. Org. Chem..

[24] Ramazani A, Kazemizadeh AR (2011). Preparation of stabilized phosphorus ylides via multicomponent reactions and their synthetic applications. Curr. Org. Chem..

[25] Kalantari F, Rezayati S, Ramazani A, Poor Heravi M (2023). Syntheses and structures of magnetic nanodendrimers and their catalytic application in organic synthesis. Appl. Organomet. Chem..

[26] Khoobi M, Foroumadi A, Emami S, Safavi M, Dehghan G, Alizadeh BH, Ramazani A, Ardestani SK, Shafiee A (2011). Coumarin-based bioactive compounds: Facile synthesis and biological evaluation of coumarin-fused 1,4-thiazepines. Chem. Biol. Drug Des..

[27] Rouhani M, Ramazani A, Joo SW (2014). Novel, fast and efficient one-pot sono-chemical synthesis of 2-aryl-1,3,4-oxadiazoles. Ultrason. Sonochem..

[28] Ali AQ, Teoh SG, Eltayeb NE, Khadeer Ahamed MB, Abdul Majid AMS, Almutaleb AA (2017). Synthesis, structure and in vitro anticancer, DNA binding and cleavage activity of palladium(II) complexes based on isatin thiosemicarbazone derivatives. Appl. Organomet. Chem..

[29] Souldozi A, Ramazani A, Dadrass AR, Ślepokura K, Lis T (2012). Efficient one-pot synthesis of alkyl 2-(dialkylamino)-4-phenylthiazole-5-carboxylates and single-crystal X-ray structure of methyl 2-(diisopropylamino)-4-phenylthiazole-5-carboxylate. Helv. Chim. Acta..

[30] Ramazani A, Mahyari AT, Rouhani M, Rezaei A (2009). A novel three-component reaction of a secondary amine and a 2-hydroxybenzaldehyde derivative with an isocyanide in the presence of silica gel: An efficient one-pot synthesis of benzo[*b*]furan derivatives. Tetrahedron Lett..

[31] Aghahosseini H, Ramazani A, Safarvand-Jalayer N, Ranjdoost Z, Souldozi A, Lepokura KŚ, Lis T (2019). Vinylphosphonium salt-mediated reactions: A one-pot condensation approach for the highly cis-selective synthesis of *N*-benzoylaziridines and the green synthesis of 1,4,2-dioxazoles as two important classes of heterocyclic compounds. Org. Lett..

[32] Bagley MC, Hughes DD, Lubinu MC, Merrit EA, Taylor PH, Tomkinson NCO (2004). Microwave-assisted synthesis of pyrimidine libraries. QSAR Comb. Sci..

[33] Kamdar NR, Haveliwala DD, Mistry PT, Patel SK (2010). Design, synthesis and in vitro evaluation of antitubercular and antimicrobial activity of some novel pyrano-pyrimidines. Eur. J. Med. Chem..

[34] Grivaky EM, Lee S (1980). Synthesis and antitumor activity of 2,4-diamino-6-(2,5-di-methoxybenzyl)-5-methylpyrido[2,3-*d*]pyrimidine. J. Med. Chem..

[35] Valderrama JA, Colonelli P, Väsquez D, Gonzälez MF, Rodríguez JA, Theoduloz C (2008). Studies on quinones Part 44: Novel angucyclinone *N*-heterocyclic analogues endowed with antitumoral activity. Bioorg. Med. Chem..

[36] Harbone J. B. (ed.) *The Flavonoids Advances in Research*, since 1980 (Chapman & Hall, London).

[37] Iacobucci GA, Sweeny JG (1983). The chemistry of anthocyanins, anthocyanidins and related flavylium salts. Tetrahedron.

[38] Bohm BA, Choy JB, Lee AYM (1989). Flavonoids of *Balsamorhiza* and *Wyethia*. Phytochemistry.

[39] Moro AJ, Parola AJ, Pina F, Pana A-M, Badea V, Pausescu I, Shova S, Cseh L (2017). 2,2′-spirobis[chromene] derivatives, chemistry, and their relation with the multistate system of anthocyanins. J. Org. Chem..

[40] Parmar VS, Jain SC, Bisht KS, Jain R, Taneja P, Jha A, Tyagi OD, Prasad AK, Wengel J, Olsen CE, Boll PM (1997). Phytochemistry of the genus *Piper*. Phytochem..

[41] Polyakov VV (1999). Chemical modification of the natural flavonoid myricetin. Chem. Nat. Compd..

[42] Grivsky EM, Lee S, Sigel CW, Duch DS, Nichol CA (1980). Synthesis, and antitumor activity of 2,4-diamino-6-(2,5-dimethoxybenzyl)-5-methylpyrido[2,3-*d*]-pyrimidine. J. Med. Chem..

[43] Esmaeilia AA, Salehan F, Habibi A, Fakhari AR (2016). Efficient synthesis of novel pyrano[2,3-*d*]pyrido[1,2-*a*]pyrimidines via isocyanide-based three-component reaction. Tetrahedron Lett..

[44] Alizadeh A, Ghanbaripour R, Ng SW (2015). An efficient synthesis of chromeno[2,3-*d*]- pyrimidine-2,4-diones with a nitroketene-aminal moiety at C(5) by a one-pot four-component reaction. Helv. Chim. Acta..

[45] Narayana Maddila S, Maddila S, van Zyl WE, Jonnalagadda SB (2015). Mn doped ZrO_2_ as a green, efficient and reusable heterogeneous catalyst for the multicomponent synthesis of pyrano[2,3-*d*]pyrimidines. RSC Adv..

[46] Brahmachari G, Banerjee B (2014). Facile and one-pot access to diverse and densely functionalized 2-amino-3-cyano-4*H*-pyrans and pyran-annulated heterocyclic scaffolds via an eco-friendly multicomponent reaction at room temperature using urea as a novel organo-catalyst. ACS Sustain. Chem. Eng..

[47] Ghorbani-Vaghei R, Maghbooli Y, Mahmoodi J, Shahriari A (2015). Poly(N-bromo-N-ethylbenzene-1,3-disulfonamide) and *N*, *N*, *N*′, *N*′-tetrabromobenzene-1,3-disulfonamide as new efficient reagents for one-pot synthesis of furano and pyranopyrimidi-nones (thiones). RSC Adv..

[48] Fan X, Wang Y, Qu Y, Xu H, He Y, Zhang X, Wang J (2011). Tandem reactions leading to bicyclic pyrimidine nucleosides and benzopyran-4-ones. J. Org. Chem..

[49] Rajesh N, Prajapati D (2015). A copper-catalyzed one-pot three-component tandem conjugative alkynylation/6-endo cyclization sequence: Access to pyrano[2,3-*d*]pyrimi-dines. Org. Biomol. Chem..

[50] Valderrama JA, Colonelli P, Vásquez D, González MF, Rodríguez JA, Theoduloz C (2008). Studies on quinones. Part 44: Novel angucyclinone *N*-heterocyclic analogues endowed with antitumoral activity. Bioorg. Med. Chem..

[51] Heber D, Heers C, Ravens U (1993). Positive inotropic activity of 5-amino-6-cyano-1,3-dimethyl-1,2,3,4-tetrahydropyrido[2,3-d]pyrimidine-2,4-dione in cardiac muscle from guinea-pig and man. Part 6: Compounds with positive inotropic activity. Die Pharm..

[52] Furuya, S., Ohtaki. T. Eur. Pat. Appl. EP. 608565, 1994, *Chem. Abstr.* **121**, 205395 (1994).

[53] Debbabi M, Nimbarte VD, Chekir S, Chortani S, Romdhane A, Jannet HB (2019). Design and synthesis of novel potent anticoagulant and anti-tyrosinase pyrano-pyrimidines and pyranotriazolopyrimidines: Insights from molecular docking and SAR analysis. Bioorg. Chem..

[54] Lee JH, Bang HB, Han SY, Jun JG (2007). An efficient synthesis of (+)-decursinol from umbelliferone. Tetrahedron Lett..

[55] Kazemi-Rad R (2018). A green approach to electrosynthesis of chromeno[3’,4’:5,6]pyrano- [2,3-*d*]pyrimidines. Anal. Methods Environ. Chem. J..

[56] Kazemi-Rad R, Azizian J, Kefayati H (2014). Electrogenerated acetonitrile anions/tetra-butylammonium cations: An effective catalytic system for the synthesis of novel chrom- eno[3′,4′:5,6]pyrano[2,3-*d*]pyrimidines. Tetrahedron Lett..

[57] Chen W, Li X, Chen L, Zhou G, Lu Q, Huang Y, Zhu W (2021). Tailoring hydro-phobic deep eutectic solvent for selective lithium recovery from the mother liquor of Li_2_CO_3_. J. Chem. Eng..

[58] Cooper MK, Downes JM, Duckworth PA, Tiekink ERT (1992). Preparation of hybrid bidentate phosphine-ligands by reduction of their benzylphosphonium or phenylphosphonium salts-X-ray crystal-structure of 2-aminophenyltriphenyl-phosphonium tetrachloronickelate(II). Aust. J. Chem..

[59] Wang H, Jing Y, Wang X, Yao Y, Jia Y (2011). Ionic liquid analogous formed from magnesium chloride hexahydrate and its physicochemical properties. J. Mol. Liq..

[60] Brahmachari G, Nurjamal K (2019). Ultrasound-assisted and trisodium citrate dihydrate-catalyzed green protocol for efficient and one-pot synthesis of substituted chromeno- [3′,4′:5,6]pyrano[2,3-*d*]pyrimidines at ambient conditions. Tetrahedron Lett..

