# Supplementary material for: An acid-based DES as a novel catalyst for the synthesis of pyranopyrimidines
Source: Sci Rep. 2023 Oct 21;13:18009. doi: 10.1038/s41598-023-45352-4 (PMC10590378; doi:10.1038/s41598-023-45352-4)

**Supporting Information**

**(Scientific Reports)**

**A****n acid-based DES as a novel catalyst for the synthesis of pyranopyrimidines**

**Arezo Monem, Davood Habibi,* Hadis Goudarzi**

Department of Organic Chemistry, Faculty of Chemistry, Bu-Ali Sina University, Hamedan 6517838683, Iran

*Corresponding author email: [davood.habibi@gmail.com](mailto:davood.habibi@gmail.com) (& dhabibi@basu.ac.ir), Tel: +98 81 38380922;

Fax: +98 81 38380709

**Content Page**

FT-IR spectrum of 2a 2

^1^H NMR spectrum of 2a 2

^13^C NMR spectrum of 2a 3

Mass spectrum of 2a 3

FT-IR spectrum of 2b 4

^1^H NMR spectrum of 2b 4

^13^C NMR spectrum of 2b 5

Mass spectrum of 2b 5

FT-IR spectrum of 2c 6

^1^H NMR spectrum of 2c 6

^13^C NMR spectrum of 2c 7

Mass spectrum of 2c 7

FT-IR spectrum of 2d 8

^1^H NMR spectrum of 2d 8

^13^C NMR spectrum of 2d 9

Mass spectrum of 2d 9

FT-IR spectrum of 2e 10

^1^H NMR spectrum of 2e 10

^13^C NMR spectrum of 2e 11

Mass spectrum of 2e 11

FT-IR spectrum of 2f 12

^1^H NMR spectrum of 2f 12

^13^C NMR spectrum of 2f 13

Mass spectrum of 2f 13

FT-IR spectrum of 2g 14

^1^H NMR spectrum of 2g 14

FT-IR spectrum of 2h 15

^1^H NMR spectrum of 2h 15

FT-IR spectrum of 2i 16

FT-IR spectrum of 2j 16

FT-IR spectrum of 2k 17

FT-IR spectrum of **2a**


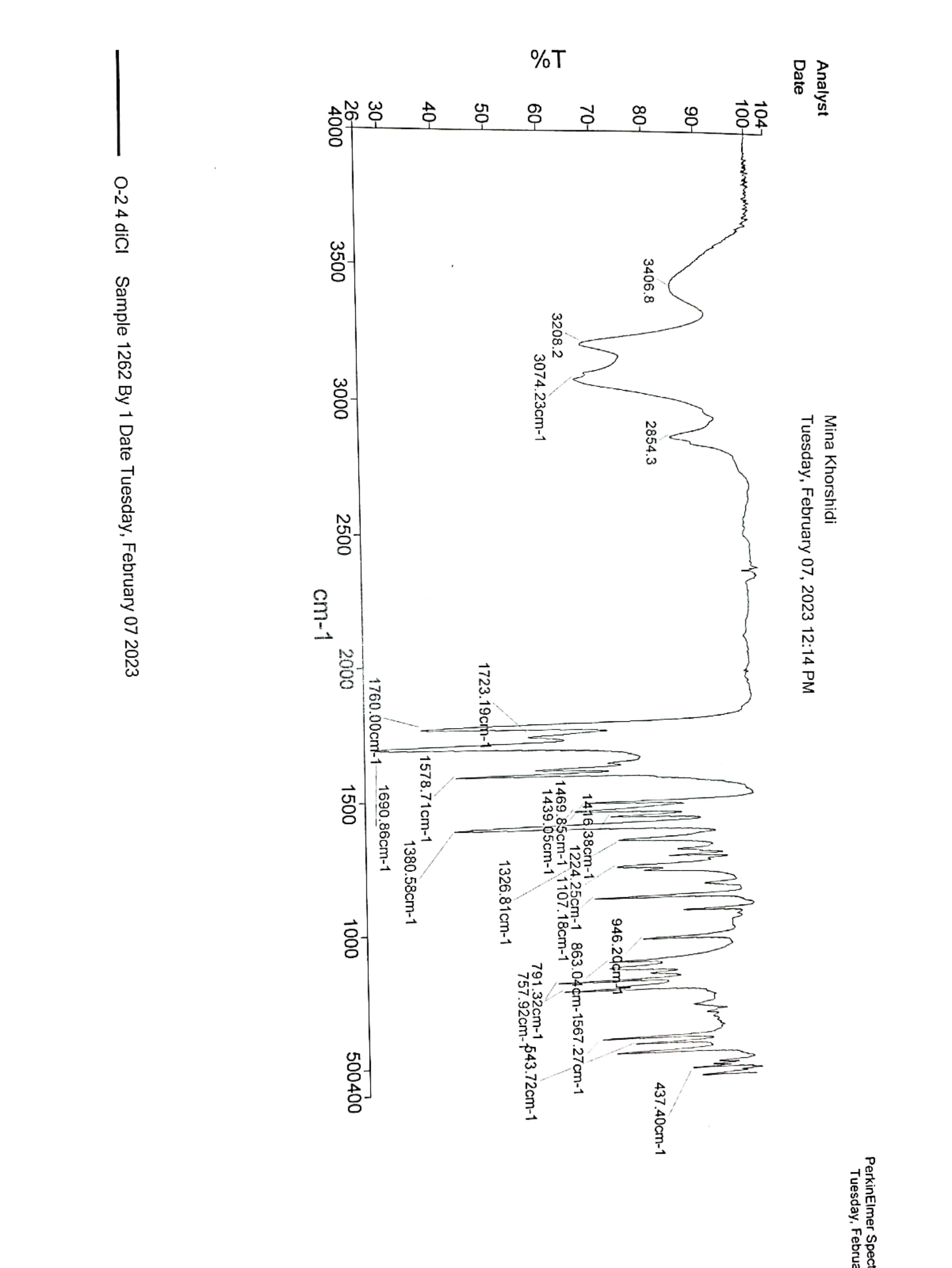


^1^H NMR spectrum of **2a**

^13^C NMR spectrum of **2a**

Mass spectrum of **2a**


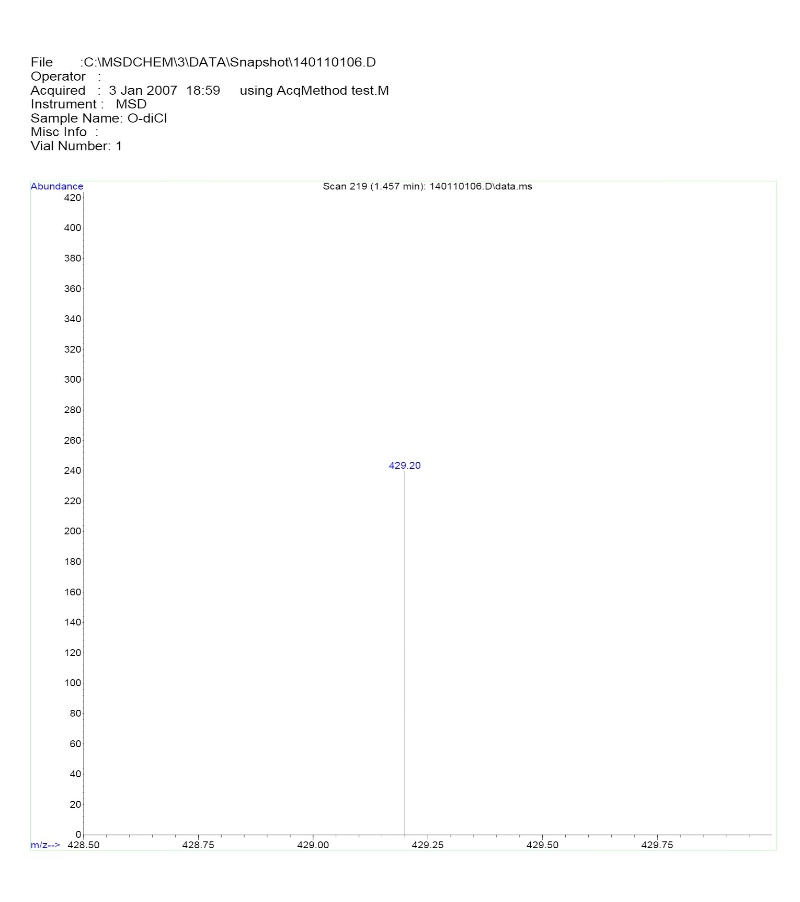

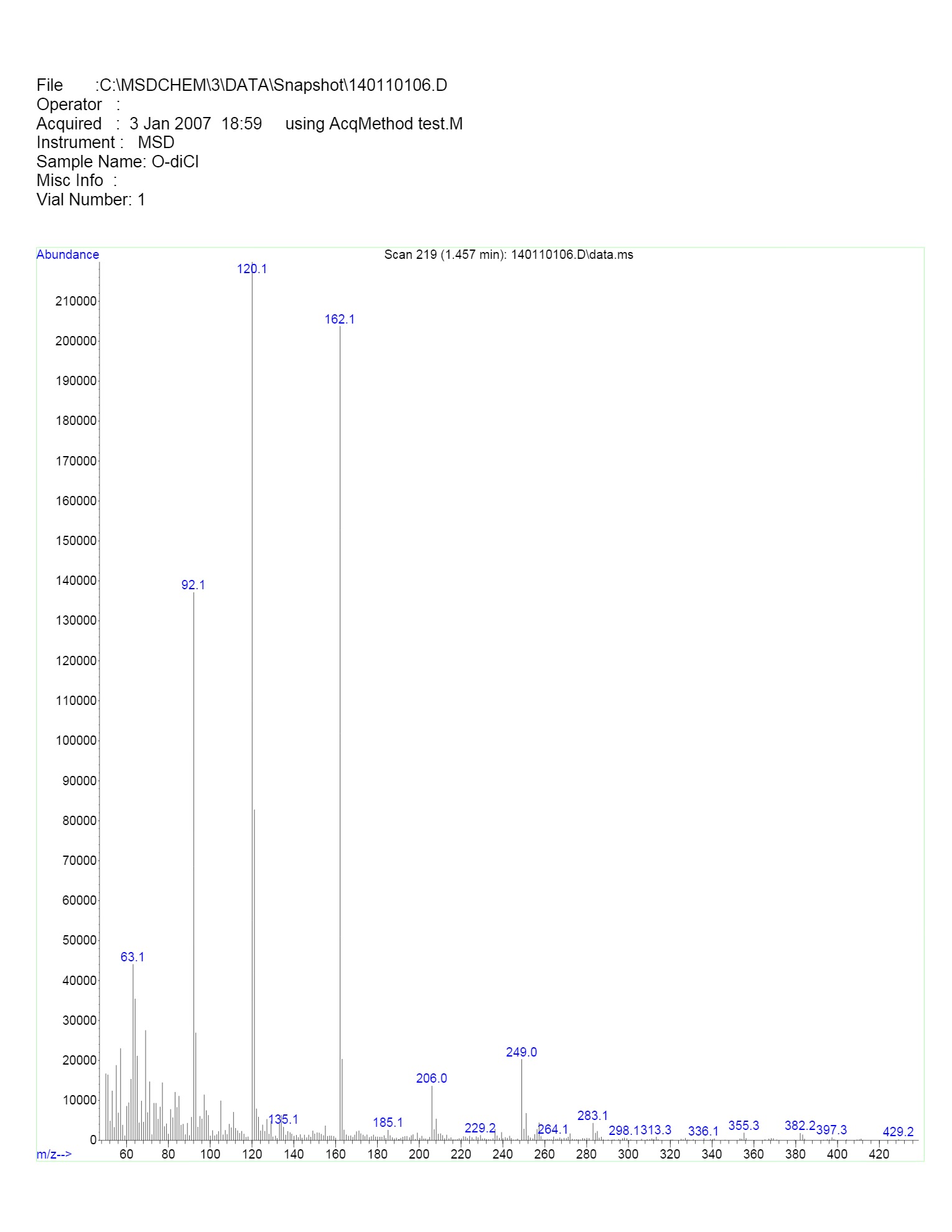


FT-IR spectrum of **2b**


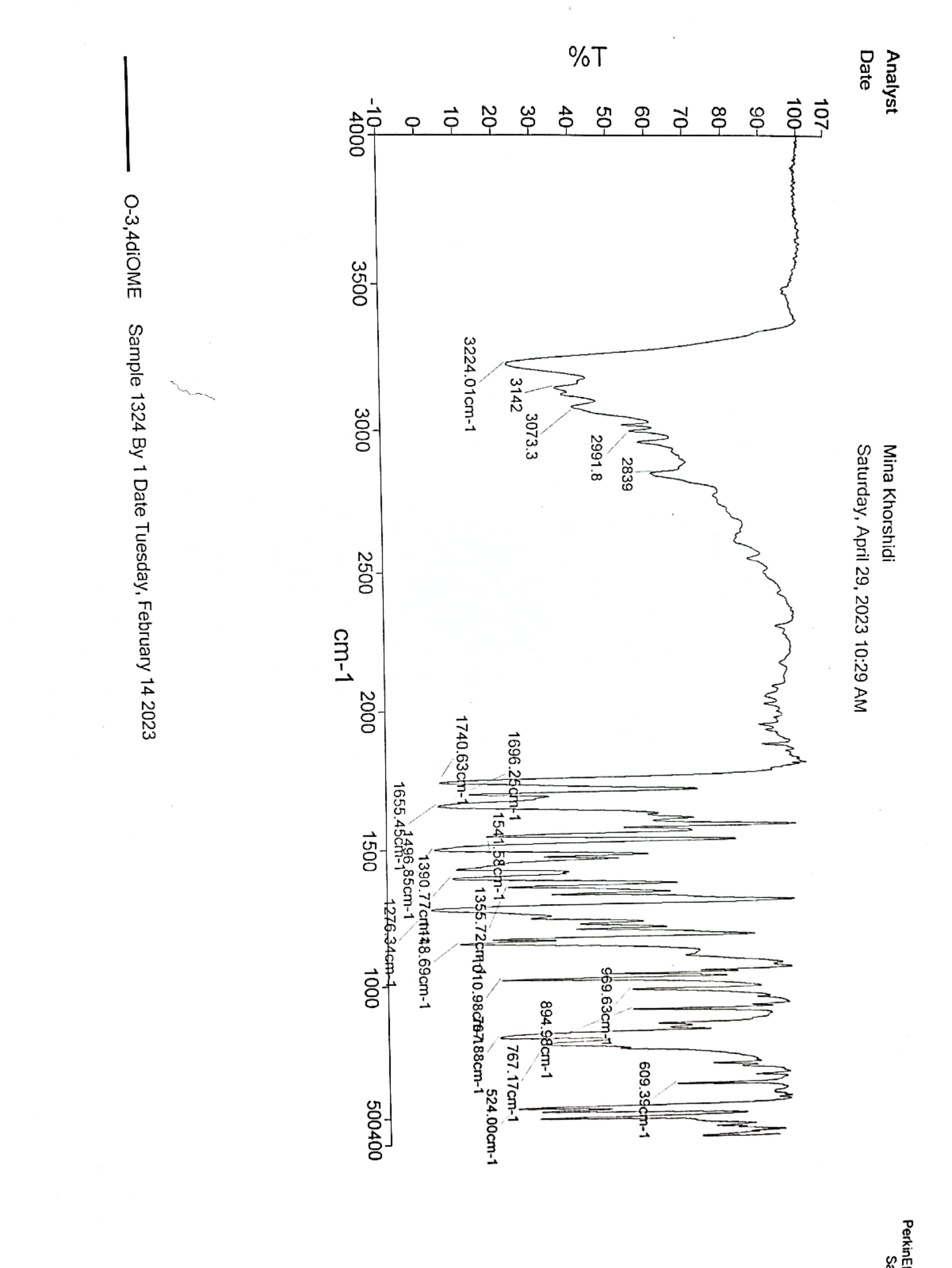


^1^H NMR spectrum of **2b**

^13^C NMR spectrum of **2b**

Mass spectrum of **2b**


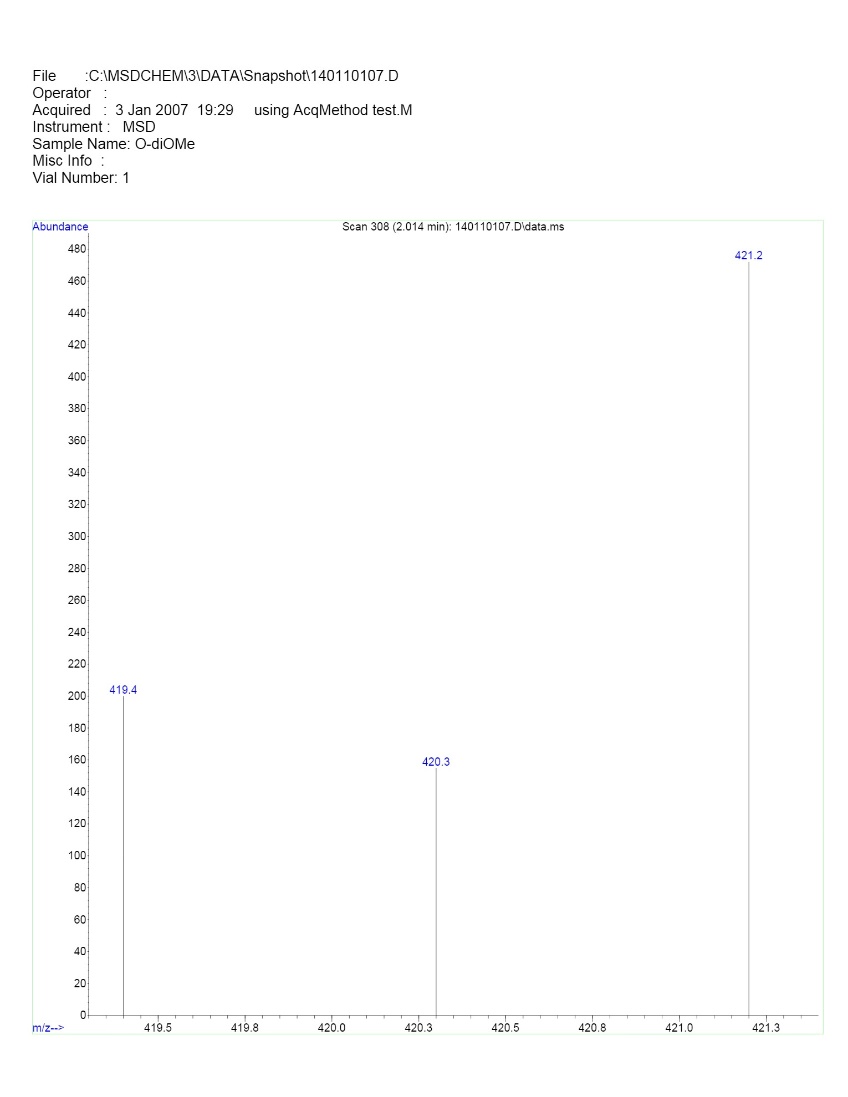

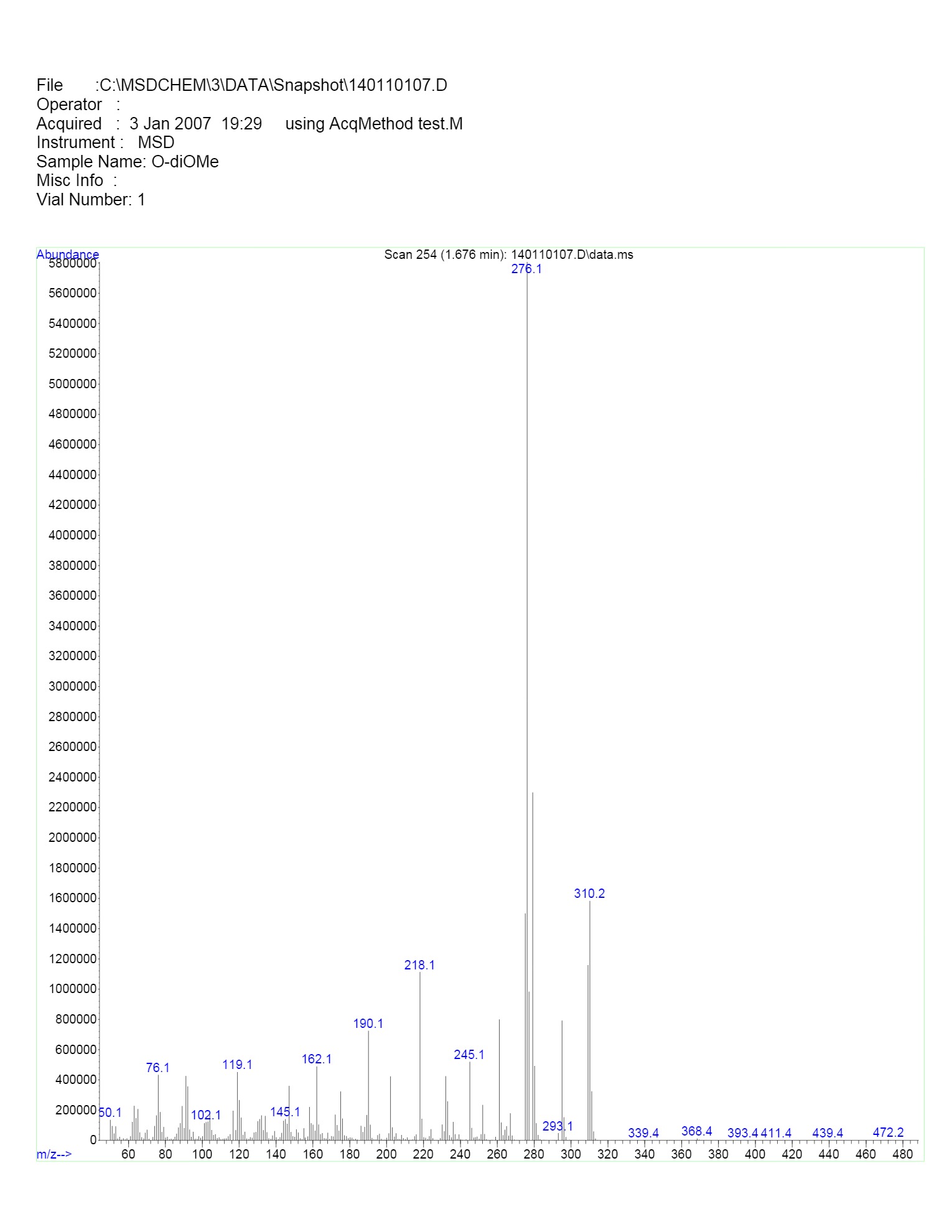


FT-IR spectrum of **2c**


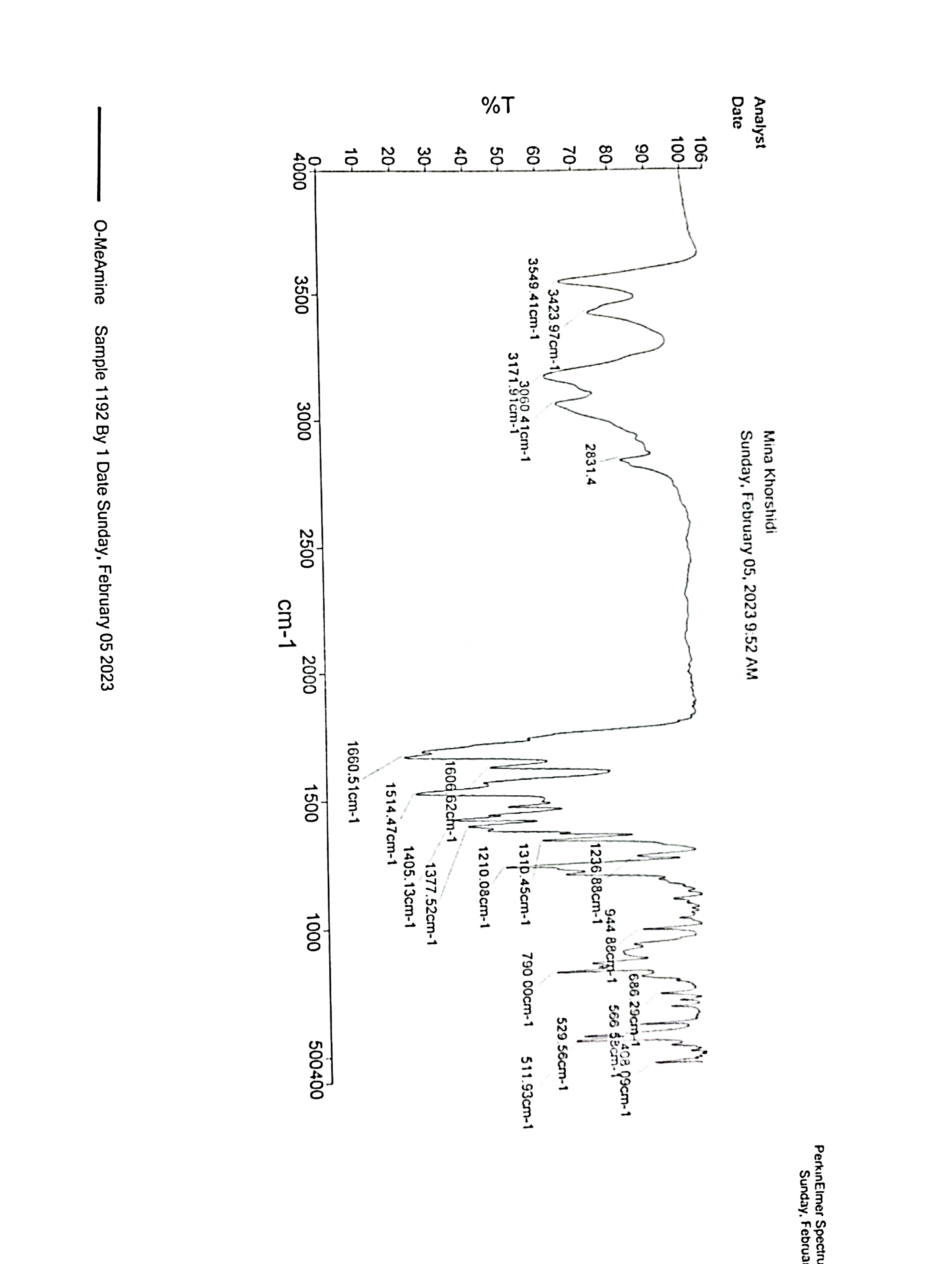


^1^H NMR spectrum of **2c**

^13^C NMR spectrum of **2c**

Mass spectrum of **2c**


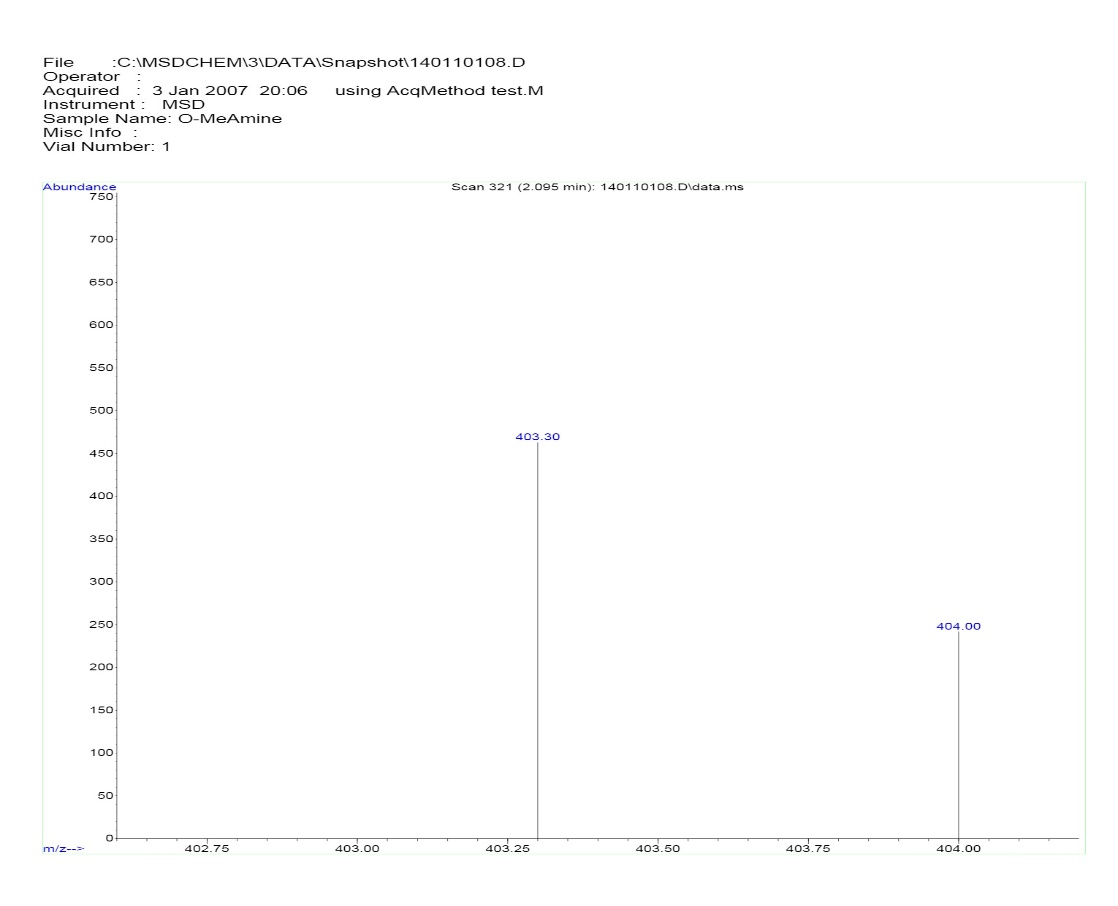

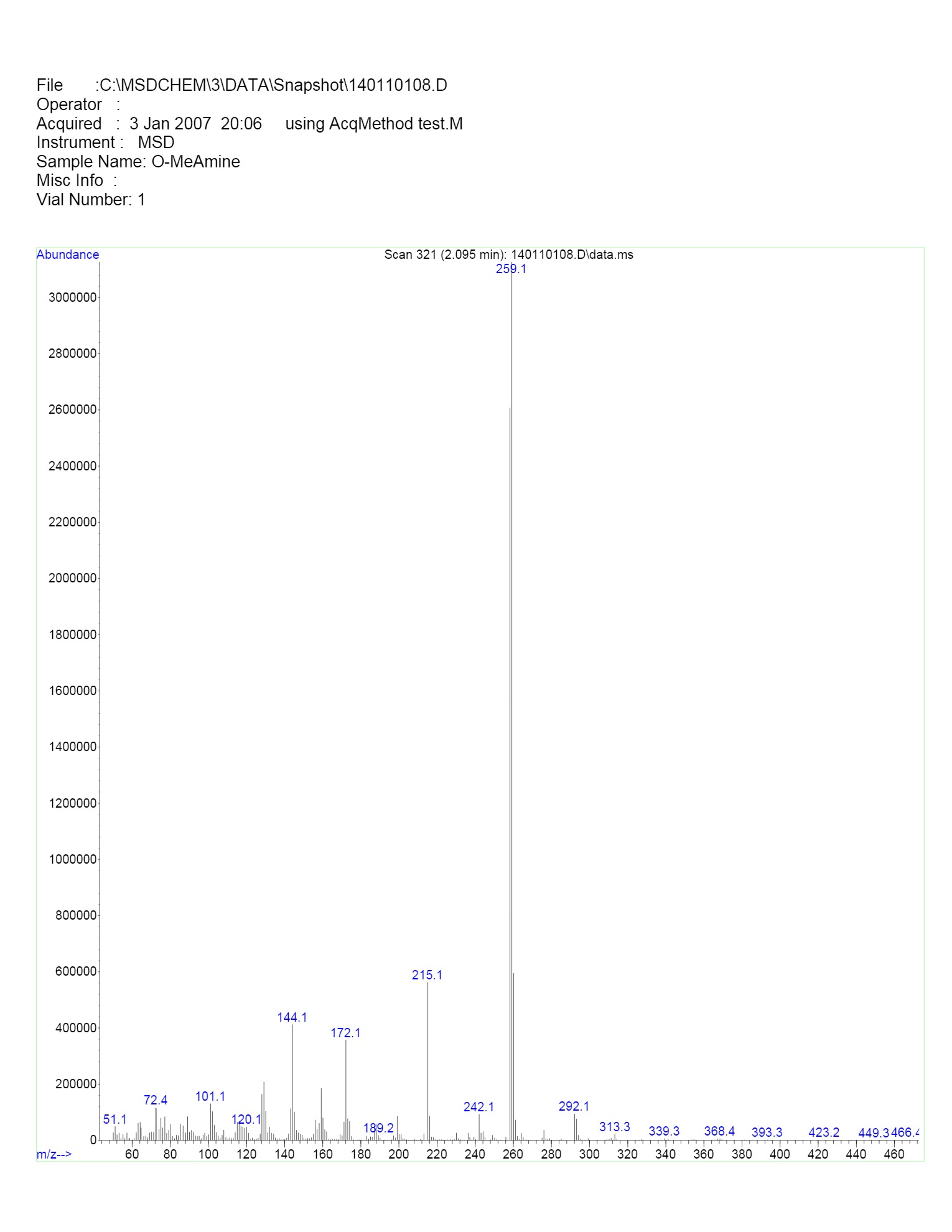


FT-IR spectrum of **2d**


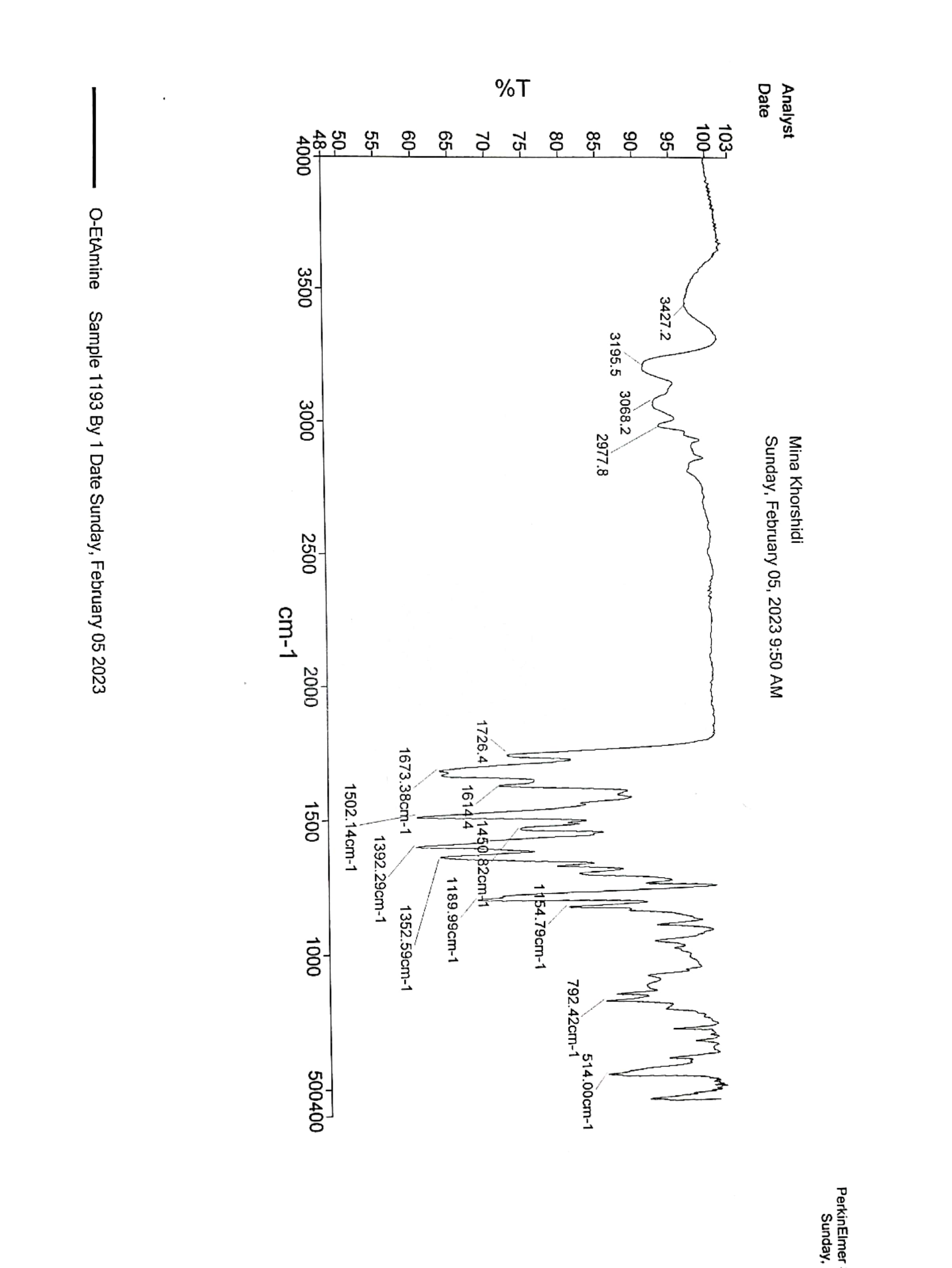


^1^H NMR spectrum of **2d**

^13^C NMR spectrum of **2d**


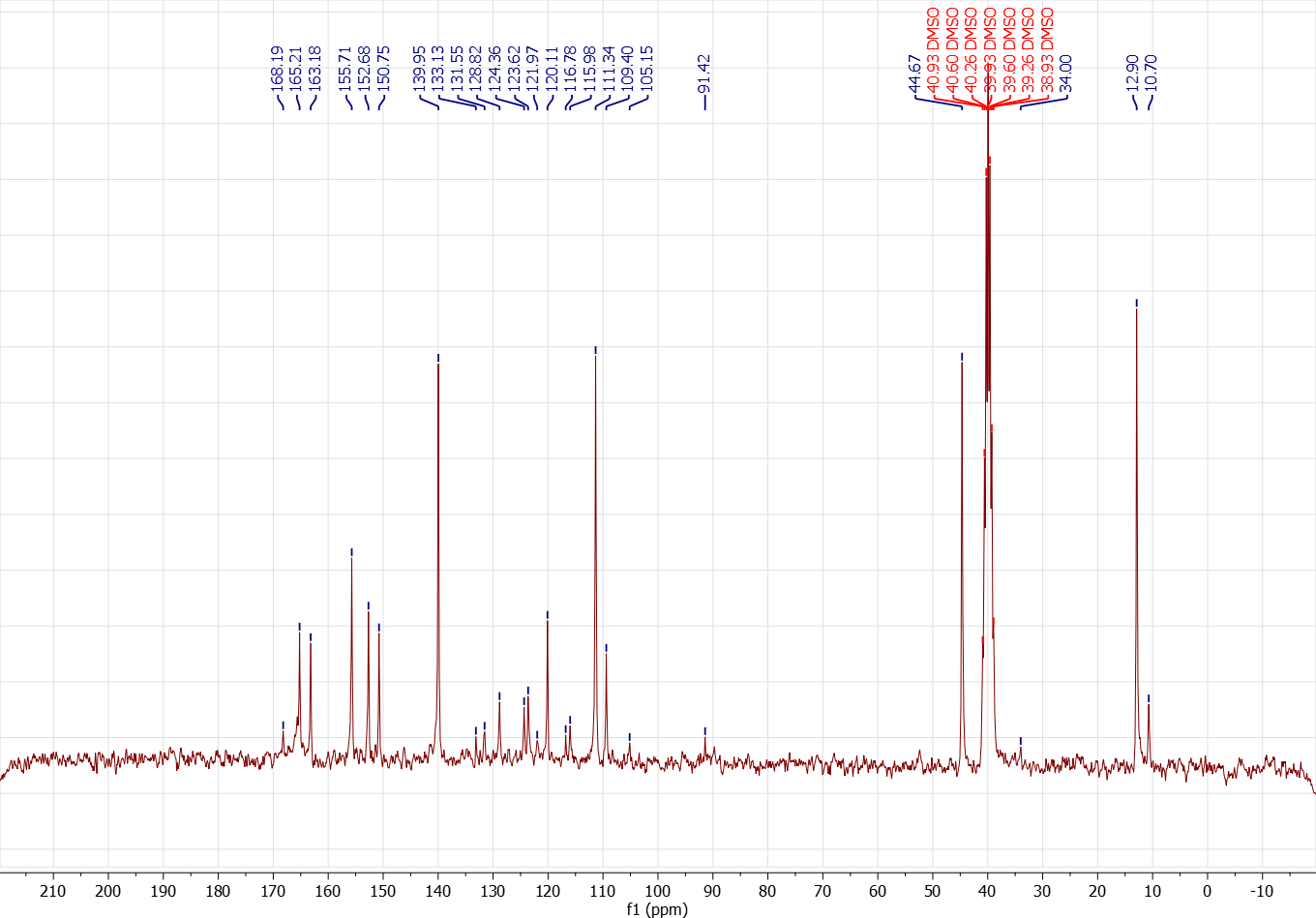


Mass spectrum of **2d**


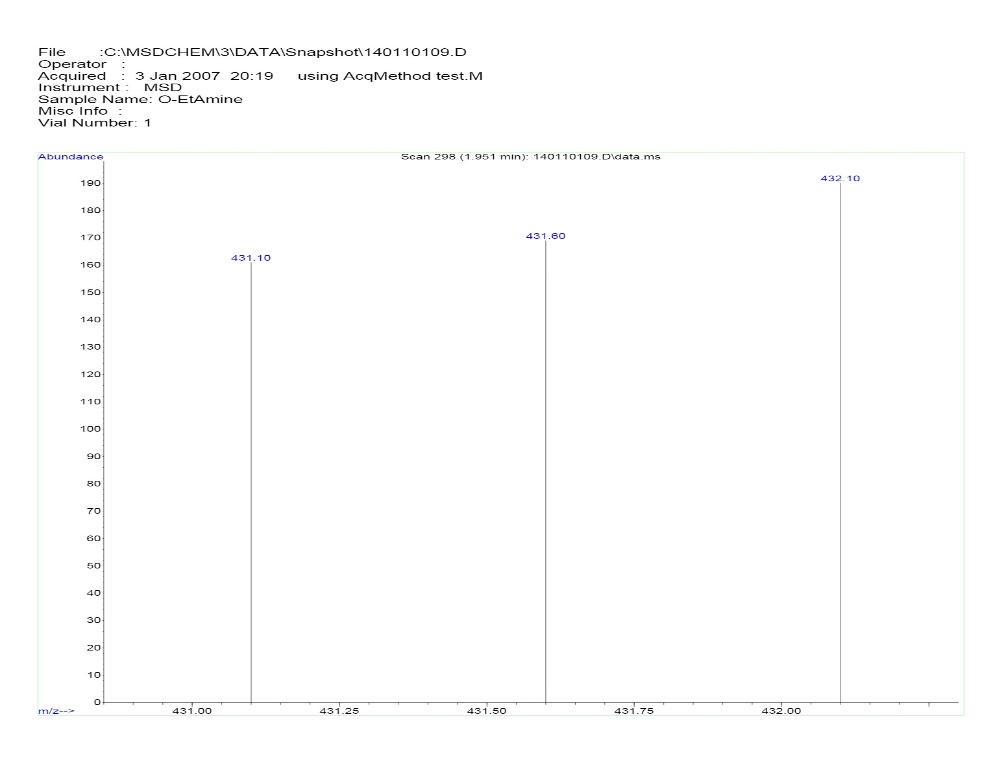

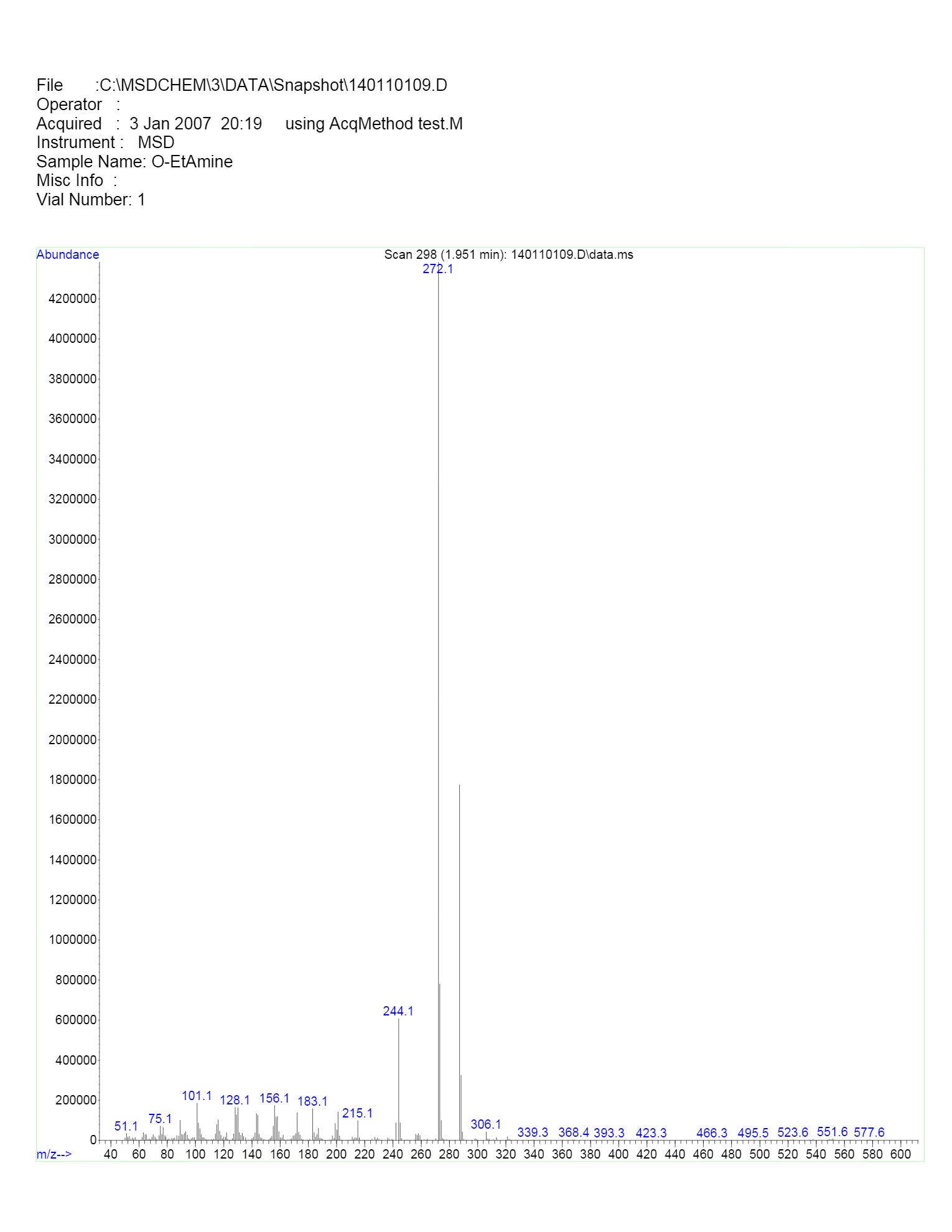


FT-IR spectrum of **2e**

^
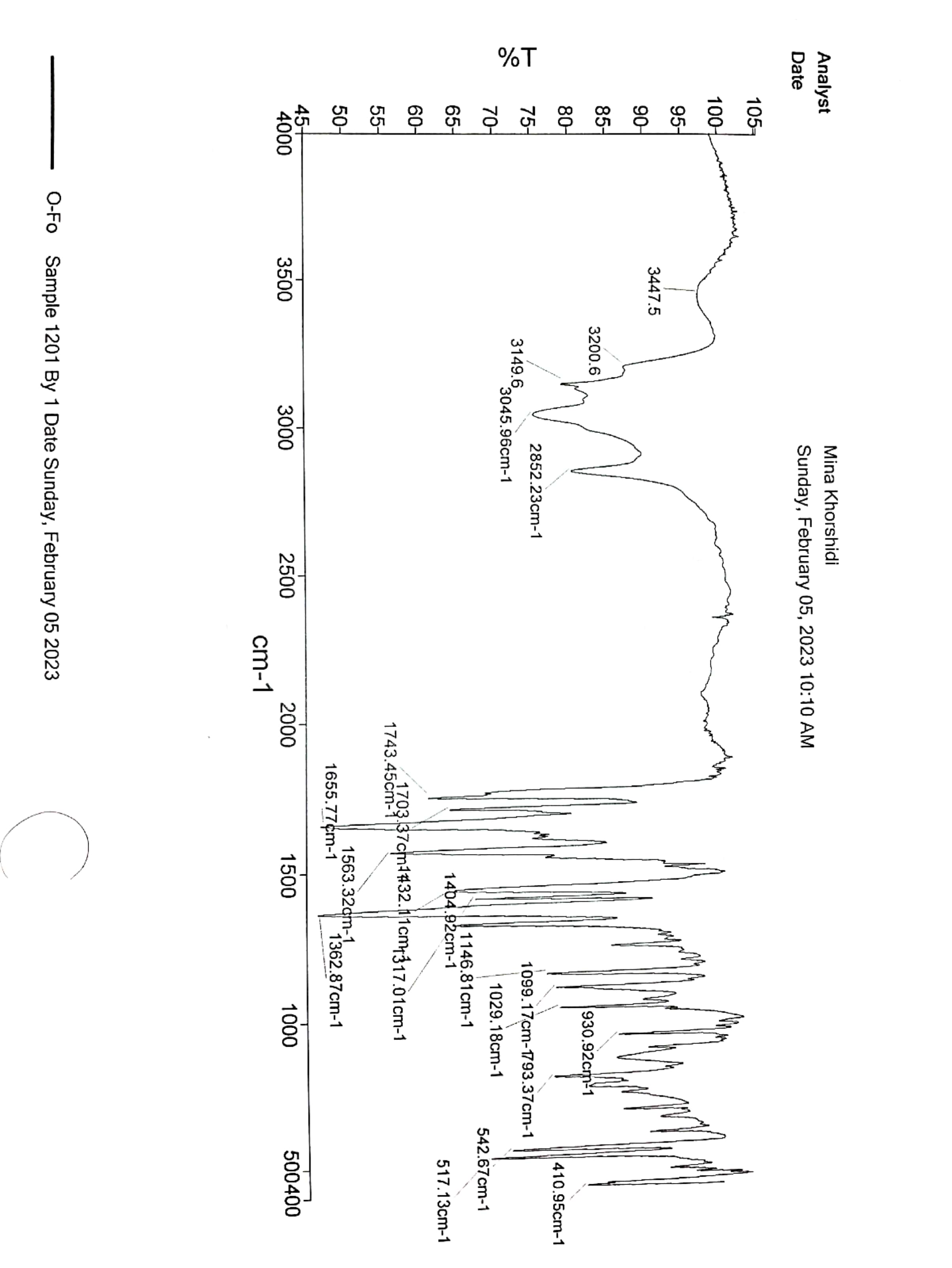
^

^1^H NMR spectrum of **2e**

^13^C NMR spectrum of **2e**


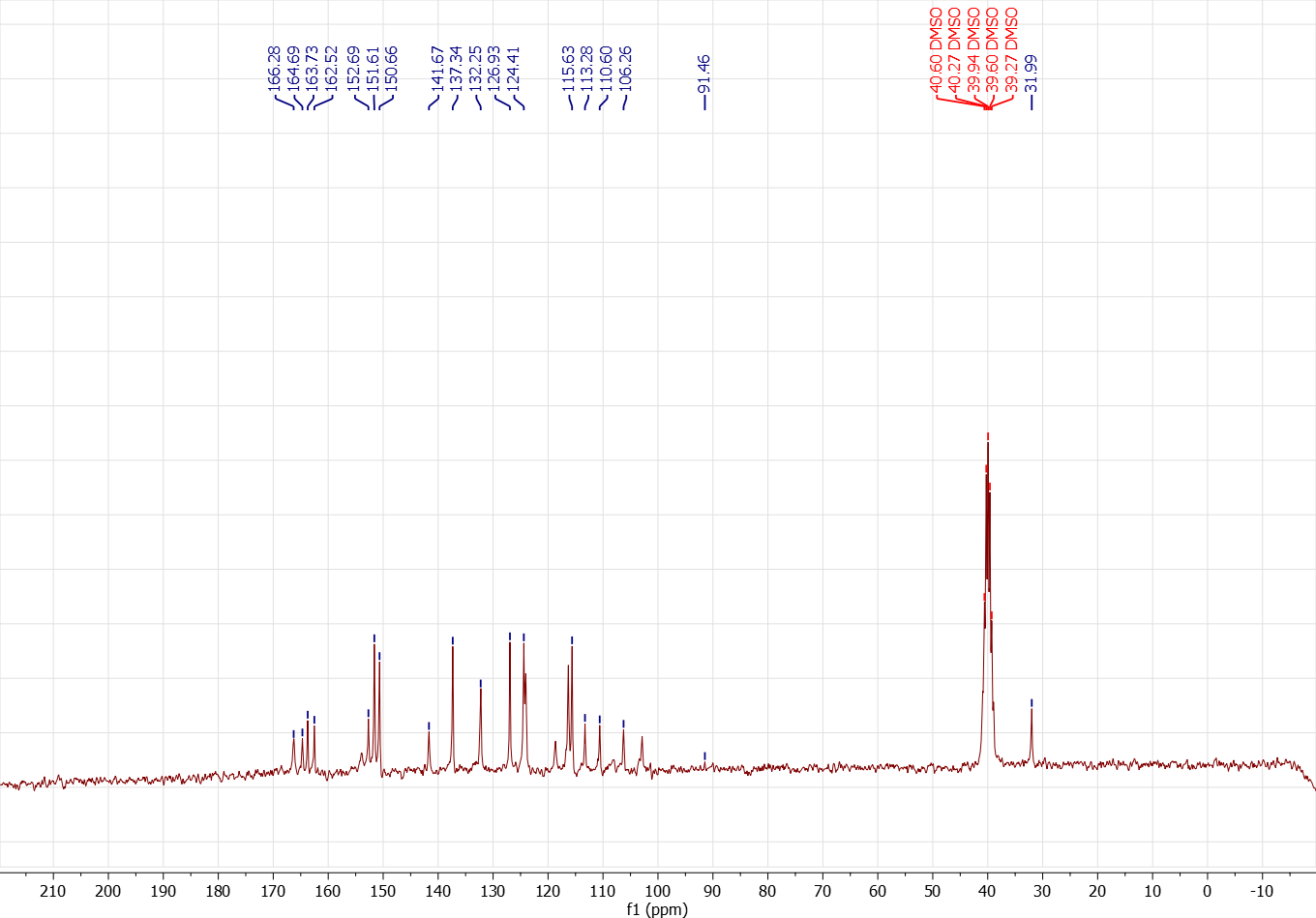


Mass spectrum of **2e**

^
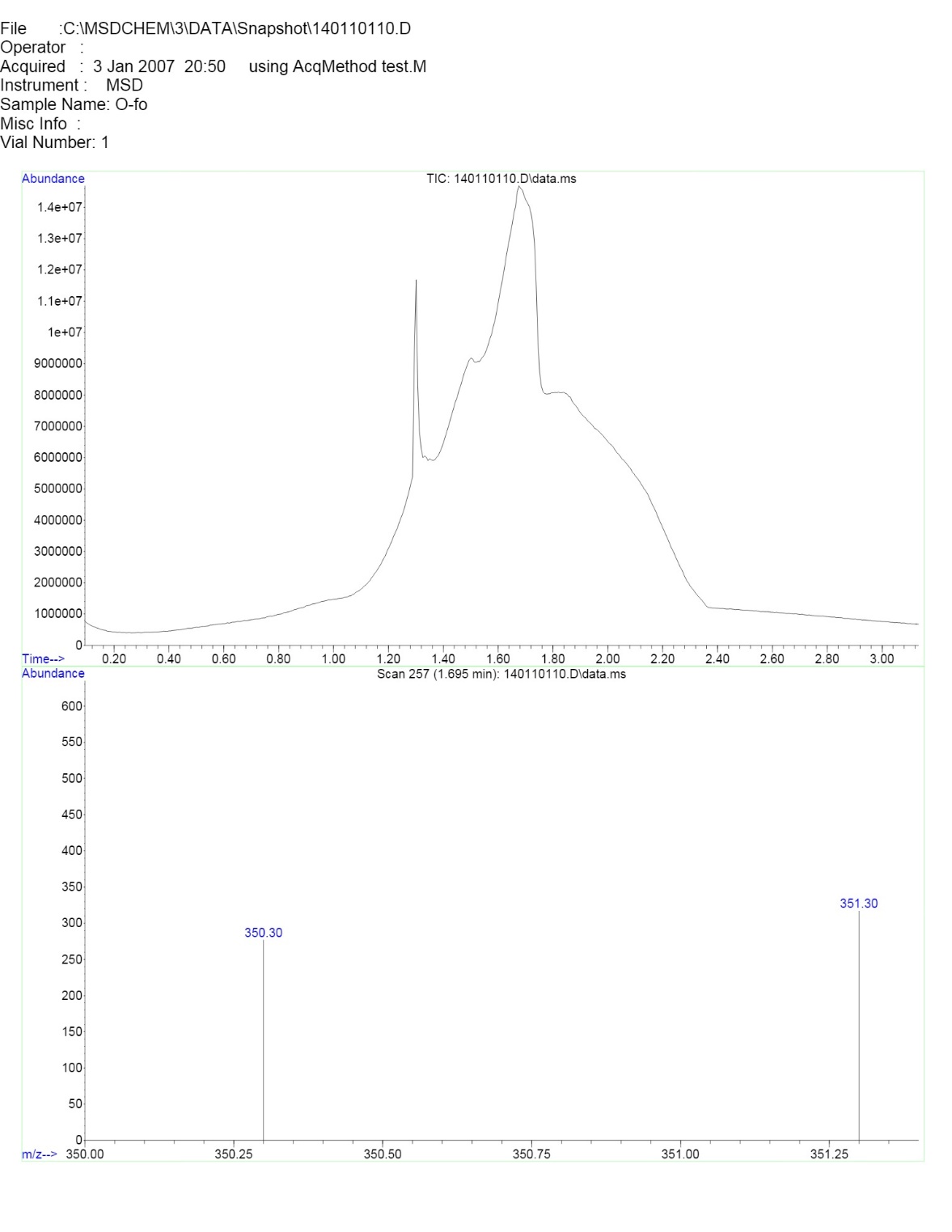
^
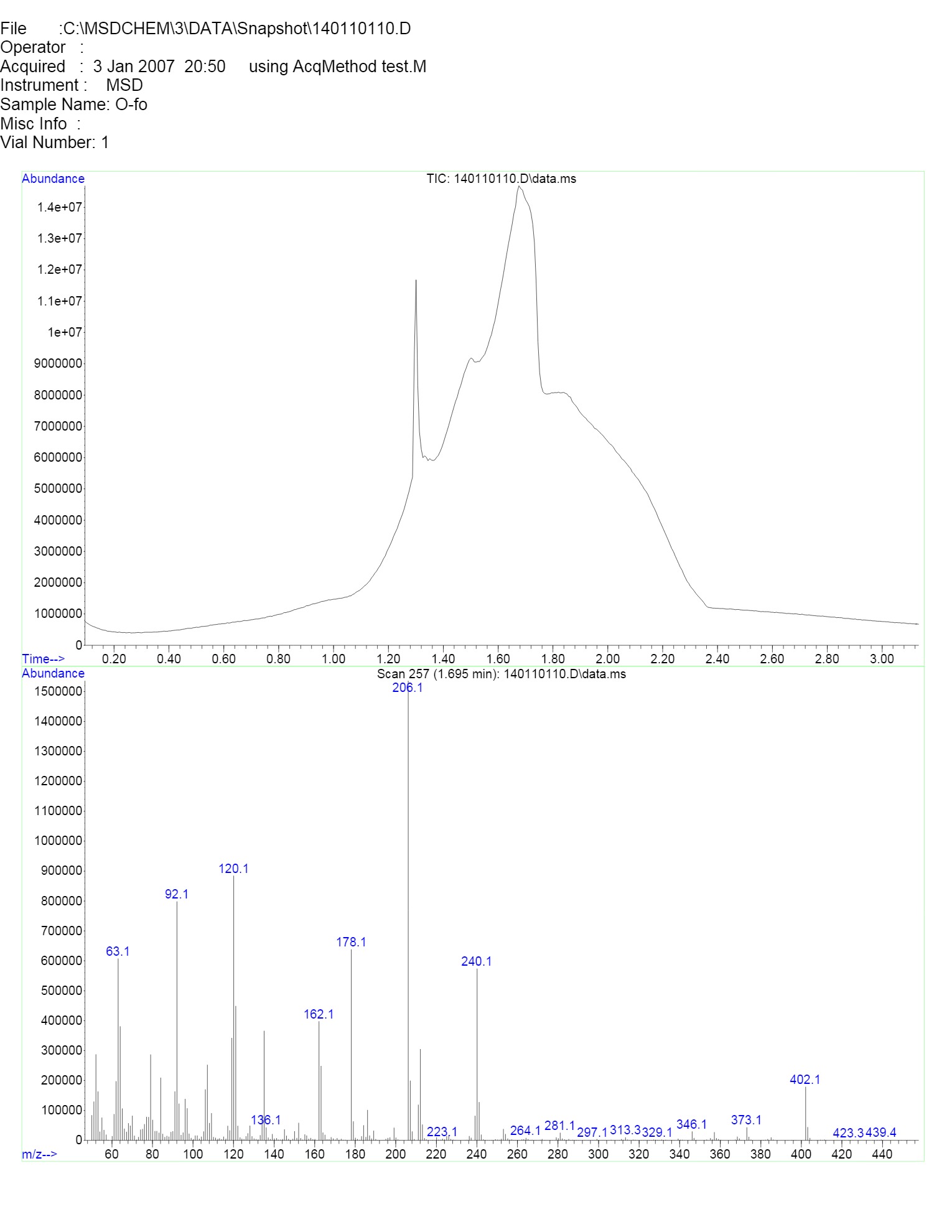


FT-IR spectrum of **2f**

^
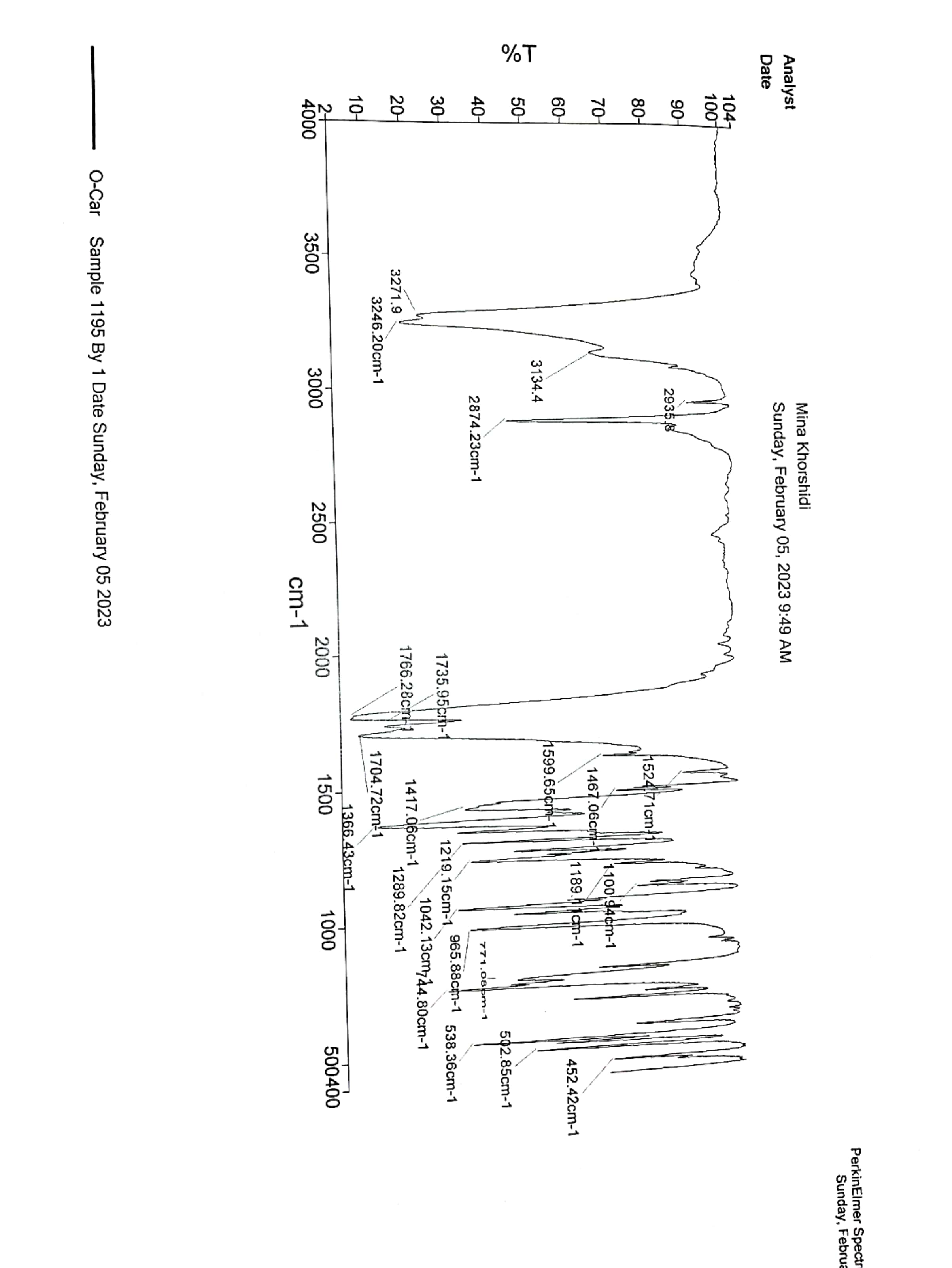
^

^1^H NMR spectrum of **2f**

^13^C NMR spectrum of **2f**

Mass spectrum of **2f**

^
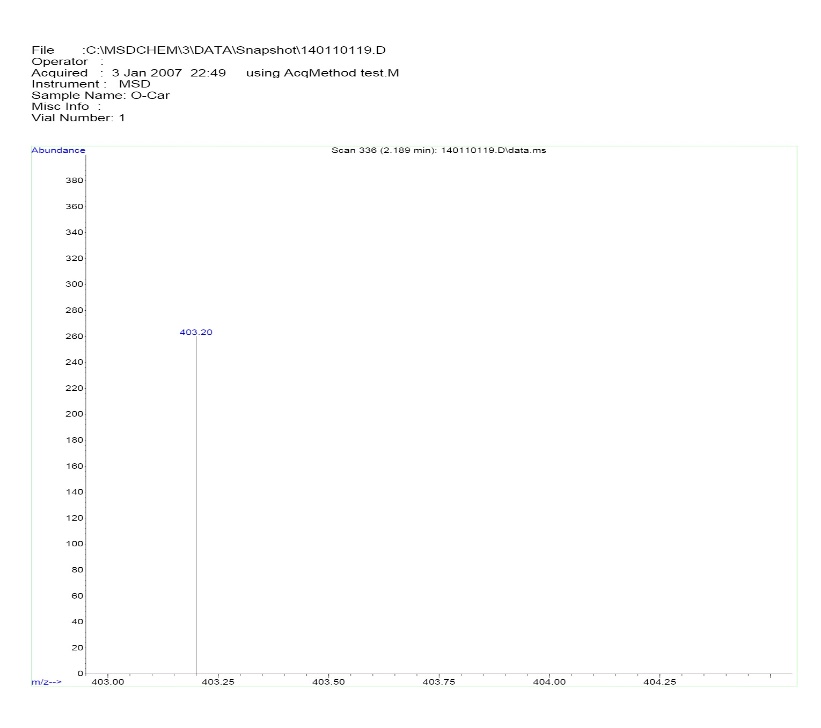
^
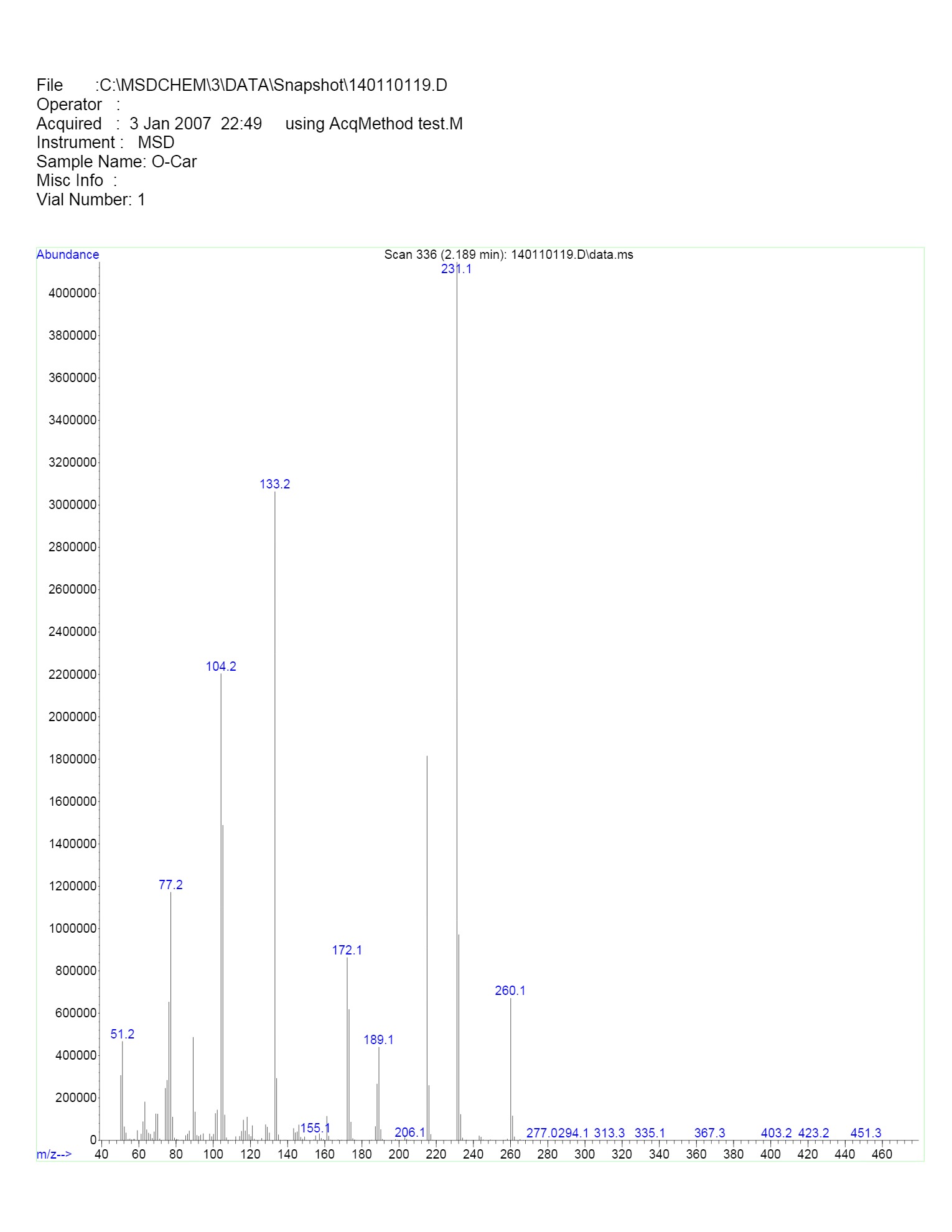


FT-IR spectrum of **2g**


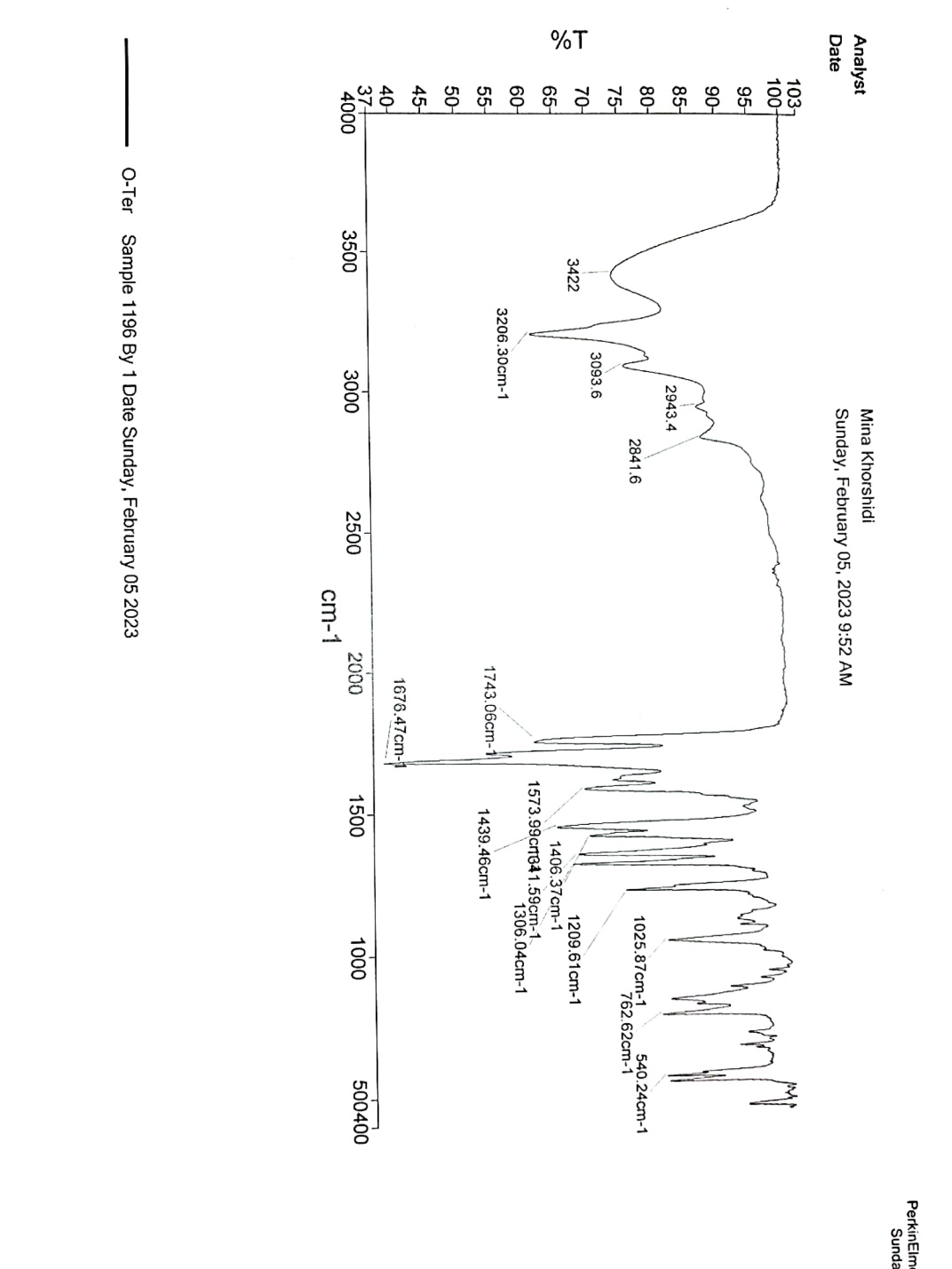


^1^H NMR spectrum of **2g**

FT-IR spectrum of **2h**

**
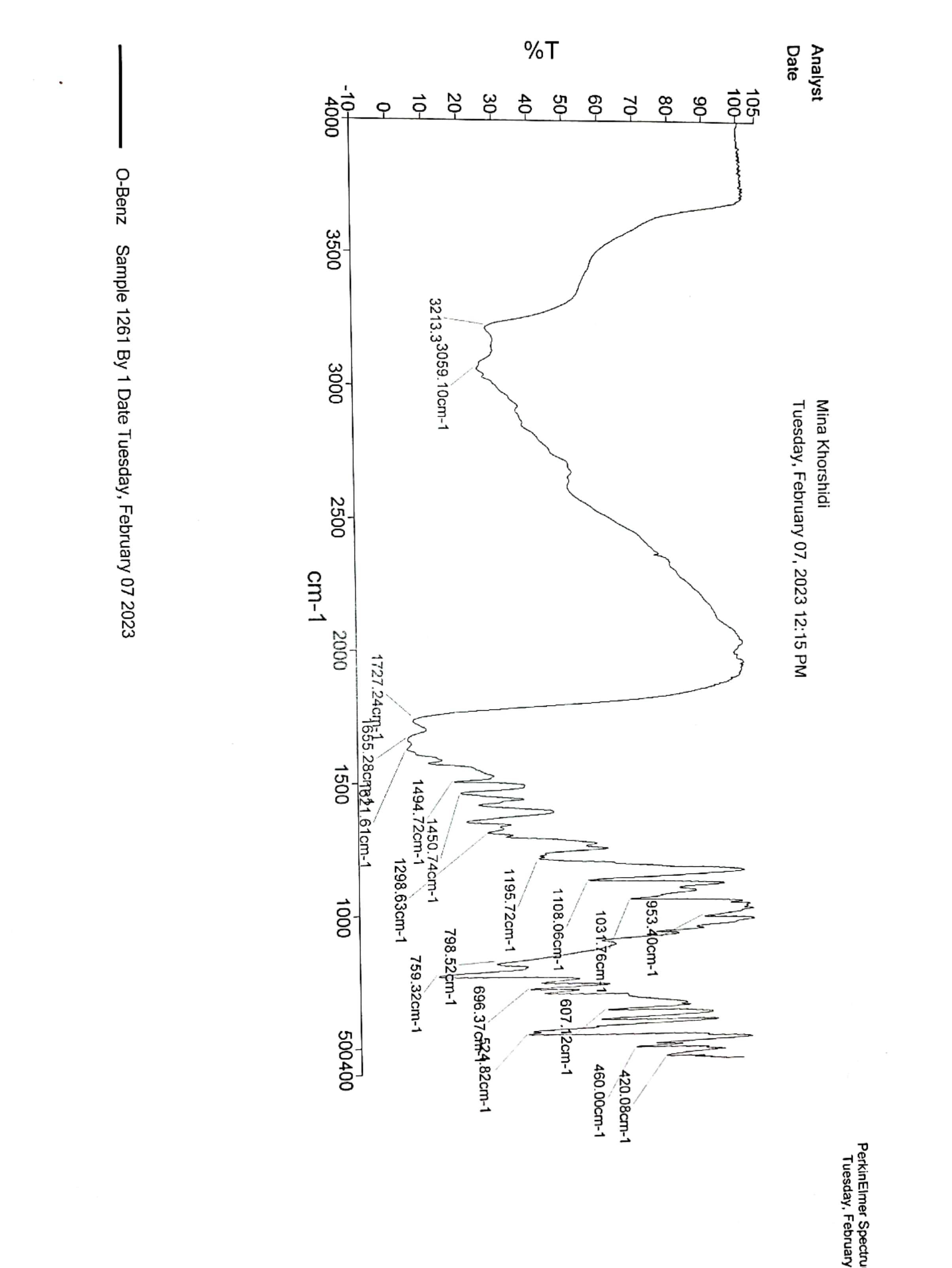
**

^1^H NMR spectrum of **2h**

FT-IR spectrum of **2i**


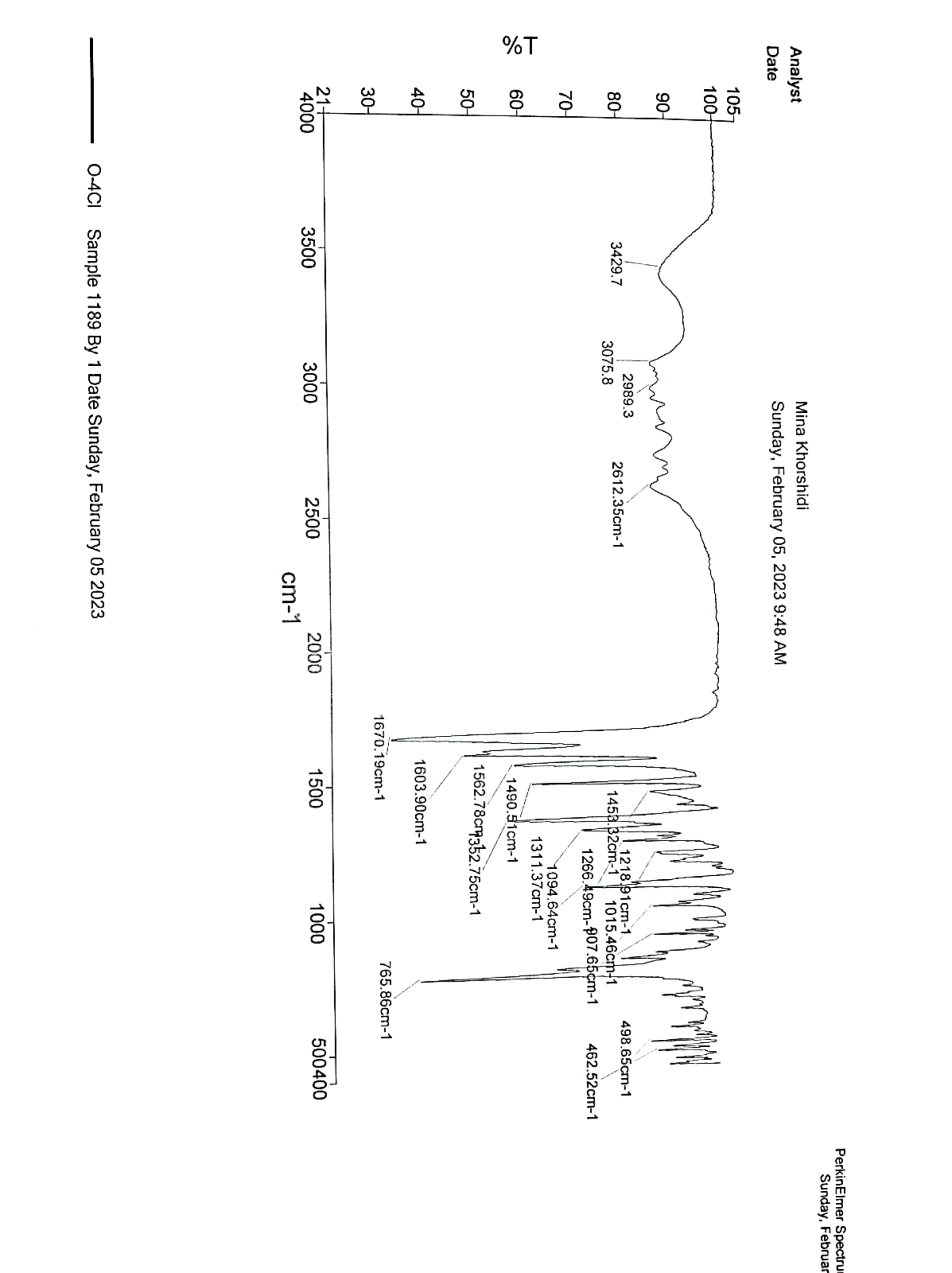


FT-IR spectrum of **2j**


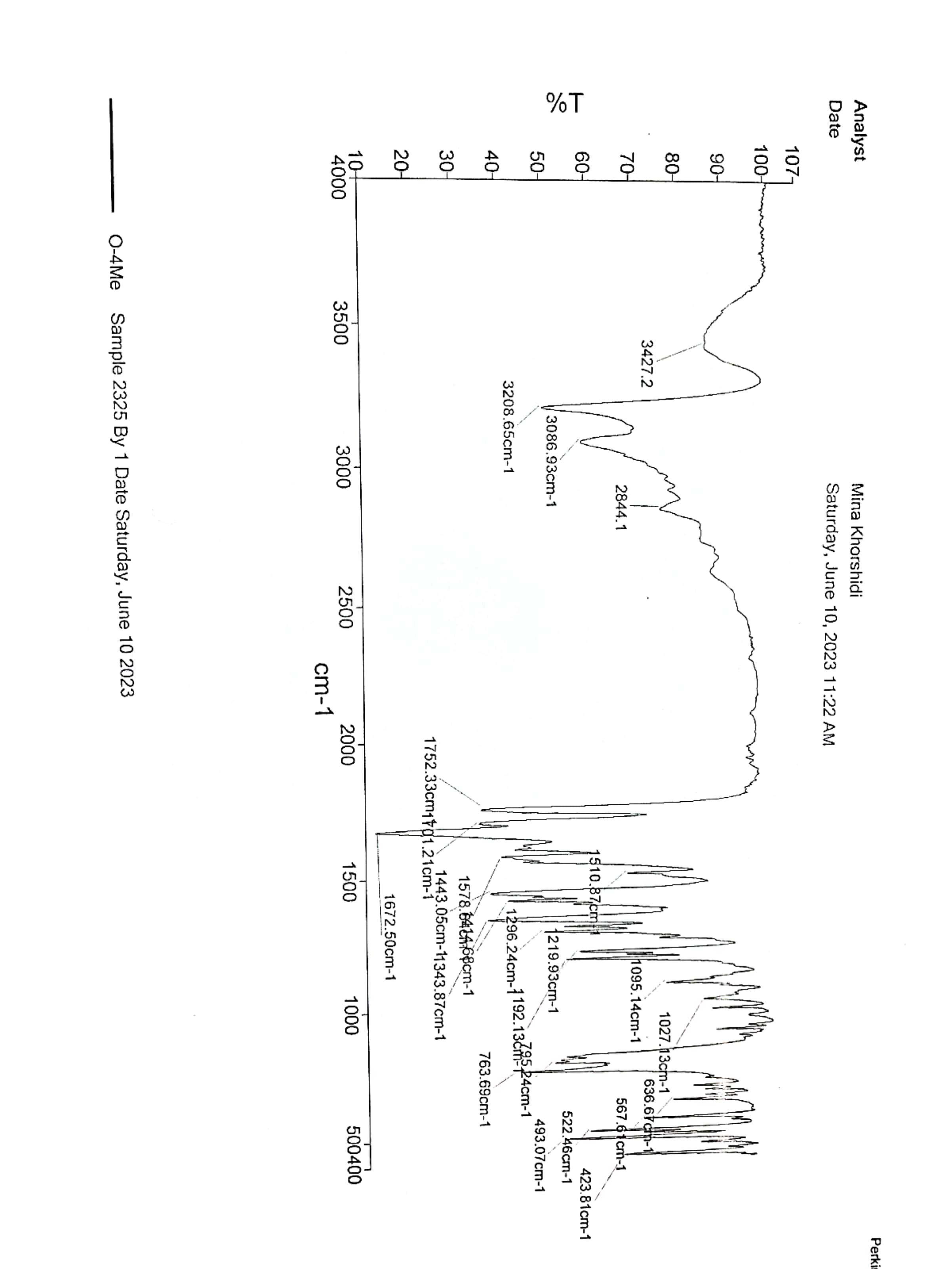


FT-IR spectrum of **2k**


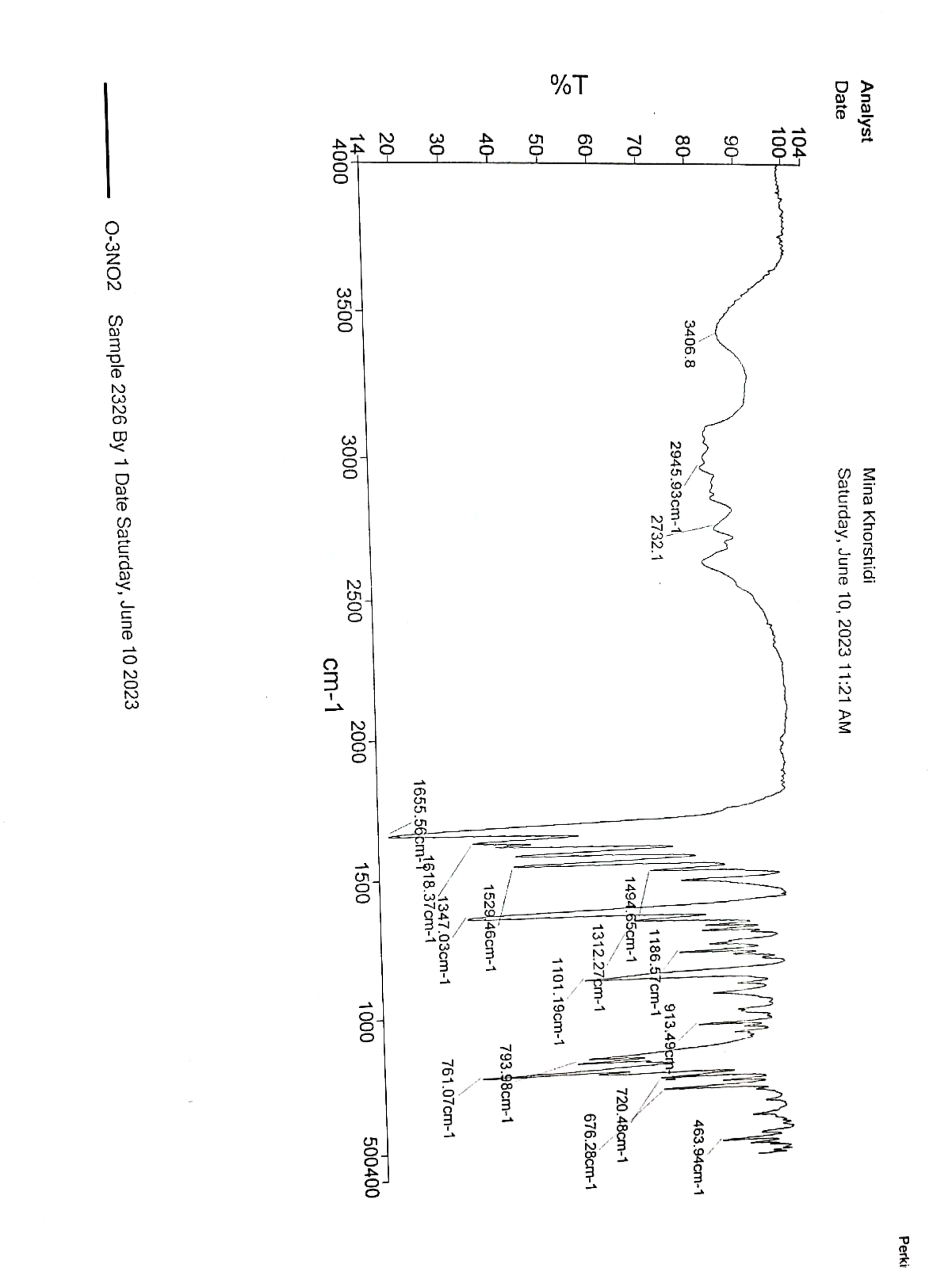

Supplement: Supplementary file 1 — Supplementary Figures. [file 41598_2023_45352_MOESM1_ESM.docx]
